# Gut metagenomic characteristics of ADHD reveal low *Bacteroides ovatus*-associated host cognitive impairment

**DOI:** 10.1080/19490976.2022.2125747

**Published:** 2022-09-20

**Authors:** Yan Li, Haiting Sun, Yufen Huang, Anqi Yin, Linjuan Zhang, Jiao Han, Yixuan Lyu, Xiangzhao Xu, Yifang Zhai, Huan Sun, Ping Wang, Jinyang Zhao, Silong Sun, Hailong Dong, Feng Zhu, Qiang Wang, Luis Augusto Rohde, Xuefeng Xie, Xin Sun, Lize Xiong

**Affiliations:** a Department of Anesthesiology and Perioperative Medicine & Center for Brain Science, The First Affiliated Hospital of Xi’an Jiaotong University, Xi’an, China; b Department of Anesthesiology and Perioperative Medicine, Xijing Hospital, the Fourth Military Medical University, Xi’an, China; c Department of Pediatrics, Xijing Hospital, the Fourth Military Medical University, Xi’an, China; d BGI-Shenzhen, Shenzhen, China; e ADHD and Developmental Psychiatry Programs, Hospital de Clínicas de Porto Alegre, Universidade Federal do Rio Grande do Sul, Brazil; f BGI-Sanya, Sanya, China; g Translational Research Institute of Brain and Brain-Like Intelligence & Department of Anesthesiology and Perioperative Medicine, Shanghai Fourth People’s Hospital Affiliated to Tongji University School of Medicine, Shanghai, China; h Shanghai Key Laboratory of Anesthesiology and Brain Functional Modulation, Shanghai, China

**Keywords:** ADHD, gut microbiota, metagenomic sequencing, microbiota-gut-brain axis, *Bacteroides ovatus*, spatial working memory, electroencephalogram rhythms, hippocampus

## Abstract

Attention-deficit/hyperactivity disorder (ADHD) is a highly heterogeneous psychiatric disorder that can have three phenotypical presentations: inattentive (I-ADHD), hyperactive-impulsive (HI-ADHD), and combined (C-ADHD). Environmental factors correlated with the gut microbiota community have been implicated in the development of ADHD. However, whether different ADHD symptomatic presentations are associated with distinct microbiota compositions and whether patients could benefit from the correction of aberrant bacterial colonization are still largely unclear. We carried out metagenomic shotgun analysis with 207 human fecal samples to characterize the gut microbial profiles of patients with ADHD grouped according to their phenotypical presentation. Then, we transplanted the candidate low-abundance bacteria identified in patient subgroups into ADHD rats and evaluated ADHD-associated behaviors and neuronal activation in these rats. Patients with C-ADHD had a different gut microbial composition from that of healthy controls (HCs) (*p* = .02), but not from that of I-ADHD patients. Eight species became progressively attenuated or enriched when comparing the compositions of HCs to those of I-ADHD and C-ADHD; in particular, the abundance of *Bacteroides ovatus* was depleted in patients with C-ADHD. In turn, *Bacteroides ovatus* supplementation ameliorated spatial working memory deficits and reversed θ electroencephalogram rhythm alterations in ADHD rats. In addition, *Bacteroides ovatus* induced enhanced neuronal activation in the hippocampal CA1 subregion. These findings indicate that gut microbial characteristics that are unique to patients with C-ADHD may be masked when considering a more heterogeneous group of patients. We link the gut microbiota to brain function in an ADHD animal model, suggesting the relevance of testing a potential bacteria-based intervention for some aspects of ADHD.

## Introduction

Attention-deficit/hyperactivity disorder (ADHD), a childhood-onset psychiatric disorder, has a worldwide prevalence of approximately 5.3% in children and adolescents and 2.5% in adults.^1,2^ ADHD is associated with highly heterogeneous impairment in cognitive and social functions and may result in poor lifetime outcomes over time, such as academic failure, mental illness and higher rates of mortality.^1,3^ According to its syndromic profiles, this neurodevelopmental disorder can be divided into inattentive (I-ADHD), hyperactive-impulsive (HI-ADHD), and combined (C-ADHD) presentations.^1,4^ Patients with the I-ADHD type exhibit atypical symptoms, including frequent inattention and disorganization; patients with the HI-ADHD type do not show inattentiveness but are restless and fidgety; and patients with the C-ADHD type exhibit high levels of both inattention and hyperactivity-impulsivity.^5^ In general, there is a progressive prevalence from HI-ADHD to I-ADHD and C-ADHD, although the proportion of ADHD subtypes has been demonstrated to be highly inconsistent in different countries and age stratifications.^6^ Importantly, the different ADHD symptomatic profiles are associated with diverse types and levels of negative outcomes.^4^ Although gene–environment interactions are implicated in ADHD development,^7,8^ little is known about the underlying pathophysiological mechanisms.

Recent studies have provided growing evidence that dysbiosis of the gut microbiota during childhood or adulthood may increase the risk of psychiatric disorders, such as major depressive disorder,^9^ bipolar disorder,^10^ schizophrenia,^11^ autism spectrum disorder,^12,13^ and ADHD.^14^ These results suggest a role of the gut microbiota in brain function and behavior and provide evidence of the communication occurring between the gut and the brain (microbiota-gut-brain axis).^15,16^ Of note, many environmental risk factors for ADHD development, such as formula feeding,^17^ antibiotic use,^18^ dietary habits,^19^ and cesarean delivery^20,^ are also associated with gut bacterial compositions.

Studies based on 16S rRNA gene amplicon sequencing have preliminarily identified that the compositions of the gut microbiota in patients with ADHD and healthy controls (HCs) are different.^21–26^ However, no studies have been carried out to compare bacterial differences between patients with different ADHD symptomatic profiles and HCs. Since there are highly heterogeneous neurobehavioral deficits among patients with different ADHD symptomatic profiles, relevant features may be missed when trying to distinguish the bacterial taxa between a mixed and heterogeneous group of patients and HCs. In addition, the 16S rRNA gene amplicon sequencing approach may omit some key information owing to the limited taxonomic and functional resolution level.

In addition, it was reported that fecal transplantation of the gut microbiota from patients with ADHD induces alterations in brain structure and function in germ-free recipient mice.^14^ There are also some probiotic interventions that are effective for reducing the risk of ADHD.^27^ However, the identity and functionality of the specific gut bacteria responsible for alleviating the abnormal behaviors of the host are still largely unknown.

Therefore, in this study, shotgun metagenomics sequencing was performed to assess the differences in the composition and function of the gut microbiota between ADHD patient subgroups. We also transplanted an attenuated bacterial species, *Bacteroides ovatus*, that was identified in patients with C-ADHD into spontaneously hypertensive rats (SHRs), an animal model of ADHD, to determine its effects on complex host behaviors and general brain function.

## Patients and methods

### Subjects

A total of 207 Chinese children and adolescents, consisting of 98 patients with ADHD (Y = 9.0 years, SD = 2.0) and 109 HCs (Y = 8.9 years, SD = 1.8), were recruited. All case and control samples were collected at Xijing Hospital, Shaanxi, China, between March 20, 2018, and February 27, 2020. Patients were diagnosed and grouped by two experienced child psychiatrists using a structured diagnostic interview conducted according to the criteria of the clinical Diagnostic and Statistical Manual of Mental Disorders, 4th edition (DSM-IV) and the guidelines of the Chinese classification of mental disorders (CCMD-3). The numbers of participants in the three different subgroups were 38 I-ADHD patients, 53 C-ADHD patients and 7 HI-ADHD patients. Patients with ADHD and HCs had to meet the following criteria: (1) age between 6 and 15 years; (2) no use of any antibiotic treatment for at least three months before sample collection; and (3) no history of treatment with any medication for ADHD. Participants who had other psychiatric or neurological diseases and any gastrointestinal or metabolic disorders were excluded. Children and adolescents whose IQs were below 70 according to the Wechsler Intelligence Scale were also excluded. All participants provided written informed consent, and the study was approved by the ethics committee of Xijing Hospital, Fourth Military Medical University (ID: KY20182002-1). The present study was registered at ClinicalTrials.gov (ID: NCT03447223).

### DNA extraction and library construction

Fresh feces samples (approximately 0.5 g) were immediately transferred into a sterilized collection tube with a sterilized wooden stick from a clean toilet by the guardians of patients and HCs. The preservation method included the use of a reagent containing imidazolium-based ionic liquid.^28^ After transport on dry ice, fecal samples were stored at −80°C until DNA extraction. Total bacterial DNA from each fecal sample was extracted from ~200 mg of stool with the NucleoSpin® Soil kit (Macherey-Nagel, Germany) according to the manufacturer’s instructions. A Qubit (Invitrogen, USA) and 1% agarose gel electrophoresis were used to analyze the quality of DNA. The details of DNA library construction were as follows: 1 μg genomic DNA was randomly fragmented to approximately 200–500 bp by sonication (Covaris, USA), and the fragmented DNA was assessed via gel-electrophotometry and then purified with an AxyPrep Mag PCR Clean-up kit (Axygen, USA; which was used to purify DNA in all steps of DNA library construction). The fragmented DNA was combined with end repair mix, incubated at 20°C for 30 min and then purified. The repaired DNAs were combined with A-tailing mix and incubated at 37°C for 30 min. Illumina adapters were ligated to the adenylated 3’ ends of the DNA fragments, which were then incubated at 16°C for 16 h and purified. Several rounds of PCR amplification with PCR primer cocktail and PCR master mix were performed to enrich for the adapter-ligated DNA fragments. The final DNA libraries were assessed for the average insert size using an Agilent 2100 Bioanalyzer (Agilent Technologies, USA) and quantified by an ABI StepOnePlus Real-Time PCR system (Applied Biosystems, USA).

### Shotgun metagenomic sequencing and microbial community profiling

Samples were sequenced on the Illumina HiSeq X Ten platform with an insert size of 300 bp (paired end, 150 base pairs). Before further bioinformatic analysis, raw reads containing adapter sequences, low-quality reads (lower Q-score 20 rate more than 50%) and ambiguous bases (N base rate more than 5%) were filtered out with SOAPnuke,^29^ and 1,486.5 Gb of high-quality PE reads were acquired for the 207 samples, with an average of 7.18 Gb per sample. To remove contamination from human host DNA, reads were aligned to the human genome reference (hg19) by SOAPaligner (v2.22, parameters: -m 280 -x 420 -r 1 -l 32 -s 75 -c 0.9),^30^ and the mapping reads were discarded. The average rate of host contamination was 1.25 ± 5.23%.

The profile of the microbial composition for each sample was calculated using MetaPhlAn2 (v2.0),^31^ which uses ~1 M unique clade-specific marker genes (including bacterial, archaeal, viral and eukaryotic) to estimate the relative abundances of bacterial taxa. The parameters of MetaPhlAn2 were set as ‘–nproc 10 – stat avg_g – ignore_viruses – ignore_eukaryotes – ignore_archaea’. Then, all sample profiles were merged using merge_metaphlan_tables.py.

Functional profiling for each sample was performed using HUMAnN2 (v0.11.2).^32^ In brief, HUMAnN2 was used to rapidly identify the known microbial species in the samples with MetaPhlAn2 and then to establish a customized pangenome database in which all genomes were preestablished and functionally annotated. Sample reads were mapped to this database with Bowtie2,^33^ with the unmapped reads translated and mapped to a protein database (UniRef90)^34^ with Diamond.^35^ Finally, all mapping reads were used to estimate gene family abundance and then annotated to metabolic enzymes to reconstruct and quantify metabolic pathways (MetaCyc).^36^ HUMAnN2 was run by the default parameters. All sample profiles were merged and renormalized using humann2_join_tables and humann2_renorm_table, respectively.

The metagenomic shotgun sequencing data for all samples have been deposited in the CNGB Nucleotide Sequence Archive (CNSA) under accession code CNP0000729.

### Classification models

To select biomarkers that could be used to discriminate subgroup patients and HCs, 5 trials of the 10-fold cross-validation were performed on a random forest model (randomForest 4.6–14 package) using the relative abundance profiles of all bacterial species (399 species), and the result was assessed by receiver operating characteristic (ROC) curves using the pROC package in R. The area under the curve (AUC) is a convenient tool for comparing and validating classifiers, and values of 0.90–1.00 are excellent, 0.80–0.89 are good, 0.70–0.79 are fair, and < 0.70 are poor.^37^

### Bacterial strain culture

*Bacteroides ovatus* (*B. ovatus*) ATCC 8483 was obtained from BeNa Culture Collection (Beijing, China) and cultured anaerobically with liquid thioglycolate medium (Qingdao Hope Bio-Technology Co., Ltd., Qingdao, China) at 37°C. *Escherichia coli* (*E. coli*) AM12-30 was isolated from fecal samples of healthy volunteers by Beijing Genomics Institute (Shenzhen, China) and cultured with Luria-Bertani medium under anaerobic conditions at 37°C.^38^ The OD600 was measured each day to assess the log growth phase of the bacterial strain. Then, the medium was centrifuged at 8000 rpm for 5 min to collect the bacteria, and sterile PBS was used to wash the bacteria twice. Subsequently, the prepared bacterial suspension was diluted with saline to 3 × 10^9^ cfu/ml, and 10 μl/g (body weight of rats) of oral gavage was administered with 40–50 ml/rat/day of drinking water.

### Animals and experimental design

Male SHRs (4 weeks old) were obtained from Beijing Vital River Laboratory Animal Technology Co., Ltd. and allowed to adapt to the standard housing environment (24 ± 2°C; humidity, 50 ± 10%; strict 12 h light-dark cycle) with a standard animal diet administered for a week.

For the behavioral experiments, the rats were randomly divided into three groups (7 ~ 9/group, 3 ~ 4/cage): Saline, ABX+Saline, and ABX+*B. ovatus*. Rats in the ABX+Saline and ABX+*B. ovatus* groups were treated with an antibiotic cocktail for the first 10 days and then saline or *B. ovatus* for the next 14 days via oral gavage. Rats in the saline group received a gavage with saline for a total of 24 days. The specific dosage of antibiotic was determined according to a previous study.^39^ In the first three days, rats were treated with amphotericin-B (1 mg/kg) daily by oral gavage. Then, an antibiotic cocktail (vancomycin: 50 mg/kg, neomycin: 100 mg/kg, metronidazole: 100 mg/kg, amphotericin-B: 1 mg/kg) was given daily by oral gavage for 7 days, and ampicillin (1 g/L) was supplemented in drinking water. The gavage volume was 4 ml/kg (body weight of rats). All antibiotics were purchased from Beijing Solarbio Science & Technology Co., Ltd. (Beijing, China). We prepared a fresh antibiotic mixture for gavage and drinking water every day. After 48 h, fresh saline or *B. ovatus* was given at 3 × 10^9^ cfu/ml and 10 μl/g (body weight of rats) to remodel the gut microbiota once a day for 14 consecutive days. During the behavioral experiments, saline or *B. ovatus* was further given in drinking water to maintain effective colonization. Rats were weighed daily to assess their health and calculate the gavage volume.

For the electroencephalogram (EEG) rhythm recording, rats were divided into two groups (12/group, 4/cage): saline and *B. ovatus*. Then, electrodes were implanted in the brain of each rat for future EEG detection. After one week of recovery, rats were treated with an antibiotic cocktail for 10 days and then saline or *B. ovatus* for the next 14 days via oral gavage. Saline or *B. ovatus* was further given in drinking water to maintain effective colonization during the EEG recording period.

The rat experiments were approved by the Animal Care Committee of Fourth Military Medical University (ID: 20171201). We made all efforts to minimize the number of rats used and their suffering.

### Behavioral experiments

Rats were handled each day during oral gavage to familiarize them with the operator and relax them. All rats were placed on the same rack. Behavioral testing was performed 24 h after the last gavage. Data were analyzed by professional analysis software (SMART 3.0, Panlab, Spain).

In the open field test, the rats were quickly placed in the open field (100 cm x 100 cm x 50 cm). The locomotor activity track of each rat was then recorded for 30 min, and the total movement distance and time spent in the central area were analyzed. Before the next assessment, the feces and urine were removed from the field, and the site was wiped with 75% ethanol to remove olfactory cues.

For the novel object recognition test, two identical objects (A1 and A2) were placed in the open field apparatus (100 cm x 100 cm x 50 cm). Then, the rat was placed in the open field with its back to the two objects. Each rat was allowed to explore the objects for 10 min and then was placed back into its original cage. After 24 h, the test period of the experiment was carried out. An old object (A1) and a new object (B) were placed in the target area, and each rat was allowed to explore the objects for 10 min. The time spent exploring the old object and new object was recorded, and the recognition index was calculated. The recognition index equals the time spent exploring object B/(time spent exploring object A1 + time spent exploring object B).

In the marble burying test, the rat was placed in a cage (25 cm x 40 cm x 18 cm) with a fresh pad of shavings (depth 5 cm) for 30 min. Then, the feces and urine were removed from the cage. The black marbles (25 mm in diameter, 20 balls/cage) were placed neatly on the padding in the same cage. The rat was allowed to explore the cage for another 30 min. Finally, two observers who were blinded to the groups were invited to count the number of buried marbles (>50% marble covered by bedding material).

In the Y Maze assessment, a Y-shaped apparatus with three black plastic arms, which marked A/B/C at a 120° angle from each other, was used, and the size of each arm was 50 cm x 16 cm x 42 cm. The rat was placed at the end of any arm and allowed to freely explore for 8 min. The camera system recorded the total number of entries and alternations between the three arms. When a rat entered three different arms consecutively without revisiting the same arm, it was defined as one complete spontaneous alternation. Finally, the alternation percentage (the number of alternations/(the total number of entries −2) *100%) was calculated by SMART 3.0 software (Panlab, Spain).

In the elevated plus maze assessment, the rat was placed in the center of the elevated plus maze facing the open arm and allowed to freely explore for 5 min. The time spent on different arms and the number of entries into different arms were recorded and calculated by SMART 3.0 software (Panlab, Spain).

### Electroencephalogram (EEG) rhythm detection

The rats were anesthetized with isoflurane (3% for induction and 1% for maintenance) and then fixed in the stereotaxic apparatus. After removing the skin and connective tissues of the head, we implanted Type B electrodes (Jiangsu Yige Biotechnology Co., Ltd, China) into the parietal cortices of rats (AP +2.935 mm and L + 1.42 mm, AP +2.935 mm and R − 1.42 mm, AP −2.935 mm and L + 1.42 mm, AP −2.935 mm and R − 1.42 mm with respect to bregma; depth, 3 mm), and electromyography (EMG) electrode wires were implanted under the neck muscles. Then, the rats were allowed to recover for one week after the operation. The EEG recording was performed 24 h after the last oral gavage treatment. The EEG rhythms (50 Hz filtering) were recorded by Medusa (Jiangsu Yige Biotechnology Co., Ltd, China) for 2 h in each rat. Frequency bands of θ (4–7.75 Hz) and sleep were further quantified. The EEG data were analyzed by investigators who were blinded to the groups.

### Immunofluorescence staining for c-Fos+ neurons

Ninety minutes after the Y maze test, the rats were euthanized and quickly sacrificed. The whole brain was fixed in 4% paraformaldehyde overnight, which was then replaced with 30% sucrose solution for dehydration. After 3–4 days, the brain sank to the bottom of the tube. The rat brain was then embedded with compound embedding agent (Sakura, USA) at an optimally low temperature used for cutting and stored at −80°C or immediately sliced.

Coronal brain sections (30 μm) were prepared using a freezing microtome (Thermo, CryoSTAR NX50). Brain slices were washed 3 times with 1x PBS for 5 min each. Triton X-100 (0.3%) was added to the slices to induce membrane breakage at 37°C for 30 min. Then, the slices were blocked in 4% goat serum for 2 h at room temperature. The slices were incubated with the primary antibody (c-Fos, 1:500, CST#2250) at 4°C overnight and washed with PBS 3 times for 5 min each. Subsequently, the slices were incubated with the secondary antibody (anti-rabbit IgG-Alexa 555, 1:1000, CST#4413) for 2 h at room temperature and washed with PBS 3 times for 5 min each. Images were obtained with an Olympus BX53 microscope (Japan).

### 16S rRNA gene amplicon sequencing

Before and after antibiotic cocktail treatment and bacterial transplantation, the fecal samples of each rat were collected at 9 AM, placed into sterile EP tubes, and stored at −80°C immediately. Then, the samples were transported to a company for sequencing. First, the microbial community genomic DNA was extracted with an E.Z.N.A.® soil DNA Kit (Omega Bio-Tek, Norcross, GA, USA), and the quality was determined by a NanoDrop 2000 UV–vis spectrophotometer (Thermo Scientific, Wilmington, USA) and agarose gel electrophoresis. The hypervariable V3-V4 region of the bacterial 16S rRNA gene was amplified with the primer pairs 338 F (5’-ACTCCTACGGGAGGCAGCAG-3’) and 806 R (5’-GGACTACHVGGGTWTCTAAT-3’). Next, the purified amplicons were pooled for library construction and sequenced on an Illumina MiSeq PE300/NovaSeq PE250 platform (Illumina, San Diego, USA) according to standard protocols. The sequencing data were processed and analyzed by QIIME2 and R packages. The sequencing analyses were performed by Wefind Biotechnology Co., Ltd. (Wuhan, China), and further data analysis was completed at PTM Biolabs Inc. (Hangzhou, China).

### Statistical analysis

The alpha diversity of samples was estimated by the Shannon diversity index at the gene family level. Beta diversity between samples was estimated by the Bray–Curtis distance at the gene family level via the ‘vegdist’ function in the vegan R package. PERMANOVA based on the Bray–Curtis distance matrix was performed via the ‘adonis’ function from the R package vegan, and the permuted *p* value was obtained by 9,999 permutations. Supervised analysis with spare partial least-squares discriminant analysis (PLS-DA) was performed using the mixOmics package in R. Differential relative abundances of taxa and pathways were detected by the Wilcoxon rank-sum test and linear discriminant analysis (LDA) effect size (LEfSe),^40^ and the significance levels were set at a *p* value < .05 and LDA effect size > 2. All data from animal experiments are displayed as the mean ± SEM, and differences were considered significant when *p* < .05. The differences among groups were identified by 2-tailed Student’s *t* test or one-way ANOVA followed by the Tukey–Kramer post hoc test (GraphPad Prism 6.0 software).

## Results

### Human studies

#### Demographic and clinical characteristics of the recruited participants

A total of 207 children and adolescents, consisting of 98 patients with ADHD (38 I-ADHD, 53 C-ADHD, 7 HI-ADHD) and 109 HCs, were recruited. As the number of subjects with HI-ADHD was too low to permit the comparison of these patients with those of other subgroups and HCs, 38 I-ADHD, 53 C-ADHD and 109 HCs were ultimately included in the subgroup analysis. The general demographic characteristics of the recruited subjects are displayed in Table 1 (I-ADHD, C-ADHD and HC). There were no significant differences among the three groups in age, body mass index (BMI), premature birth, maternal pregnancy with metabolic disease, or antibiotic use during pregnancy or infancy. As expected, the proportion of male subjects among the C-ADHD subgroup was higher than that among HCs (98.1% vs. 81.7%, *p* = .007). The proportion of only children among the C-ADHD subgroup was lower than that among HCs (39.6% vs. 65.1%, *p* = .007). The percentage of cesarean deliveries among patients with I-ADHD was lower than that among HCs (36.8% vs. 60.6%, *p* = .023). Furthermore, as expected, low birth weight occurred more often in the C-ADHD subgroup than in HCs (20.8% vs. 5.5%, *p* = .012), and patients in the I-ADHD and C-ADHD groups had lower scores on the intelligence quotient (IQ) (*p* < .001). Predictably, the Diagnostic and Statistical Manual of Mental Disorders_Attention Deficits (DSM_AD) scores and DSM_Hyperactivity/Impulsivity Deficits (DSM_HD) scores were higher in patients with I-ADHD and C-ADHD than in HCs. Since the host characteristics were partly matched between subgroups, PERMANOVA was performed to explore the influence of these characteristics on gut microbiota (Table S1).

**Table 1.** The baseline characteristics of ADHD subgroup patients (I-ADHD = 38; C-ADHD = 53) and HCs (n = 109) in the study cohort.

Characteristics	I-ADHD (n = 38)	C-ADHD (n = 53)	HC (n = 109)	*p value
Age, years, mean ± S.D.	9.4 ± 2.1	8.8 ± 1.9	8.9 ± 1.8	0.372
BMI, kg/m^2^, mean ± S.D.	17.3 ± 3.9	16.9 ± 2.7	17.1 ± 4.3	0.737
Male, No. (%)	33 (86.8)	52 (98.1)	89 (81.7)	0.007^b^
IQ, mean ± S.D.	103.0 ± 14.5	101.9 ± 13.5	112.0 ± 13.5	4.399E-05^ab^
Only child, No. (%)	19 (50.0)	21 (39.6)	71 (65.1)	0.007^b^
Maternal pregnancy with metabolic disease, No. (%)	0 (0)	2 (3.8)	2 (1.8)	0.645
Cesarean section, No. (%)	14 (36.8)	24 (45.3)	66 (60.6)	0.023^a^
Premature birth, < 37 weeks, No. (%)	2 (5.3)	4 (7.5)	6 (5.5)	0.920
Low birth weight, < 2.5 kg, No. (%)	5 (13.2)	11 (20.8)	6 (5.5)	0.012^b^
Maternal antibiotic use during pregnancy, No. (%)	2 (5.3)	0 (0)	1 (0.9)	0.151
Antibiotic use during infancy, No. (%)	5 (13.2)	4 (7.5)	8 (7.3)	0.510
DSM_AD scores, mean ± S.D.	6.6 ± 0.9	7.5 ± 1.2	1.6 ± 1.1	4.728E-34^abc^
DSM_HD scores, mean ± S.D.	1.9 ± 1.1	7.0 ± 1.6	0.9 ± 0.9	2.010E-29^abc^

S.D., standard deviation; BMI, body mass index; IQ, intelligence quotient; DSM, Diagnostic and Statistical Manual of Mental Disorders; DSM_AD, DSM attention deficits; DSM_HD, DSM hyperactivity/impulsivity deficits. *p value based on the Kruskal–Wallis test (continuous variables, or the Wilcoxon rank-sum test for two groups) or Fisher’s exact test (categorical variables) for all groups. ^a^*p* < 0.05 for I-ADHD and HC; ^b^*p* < 0.05 for C-ADHD and HC; ^c^*p* < 0.05 for I-ADHD and C-ADHD.

The general dietary and defecation habits among the I-ADHD, C-ADHD and HC groups are listed in Table S2 and Table S3. We found no significant differences among the three groups in any of the dietary or defecation habits, including infant feeding; the preference for side dishes, staple foods, yogurt, and other fermented food; and frequency, smoothness, and shape of bowel movements.

#### Fecal microbiome diversity in all patients with ADHD

**Figure 1.**
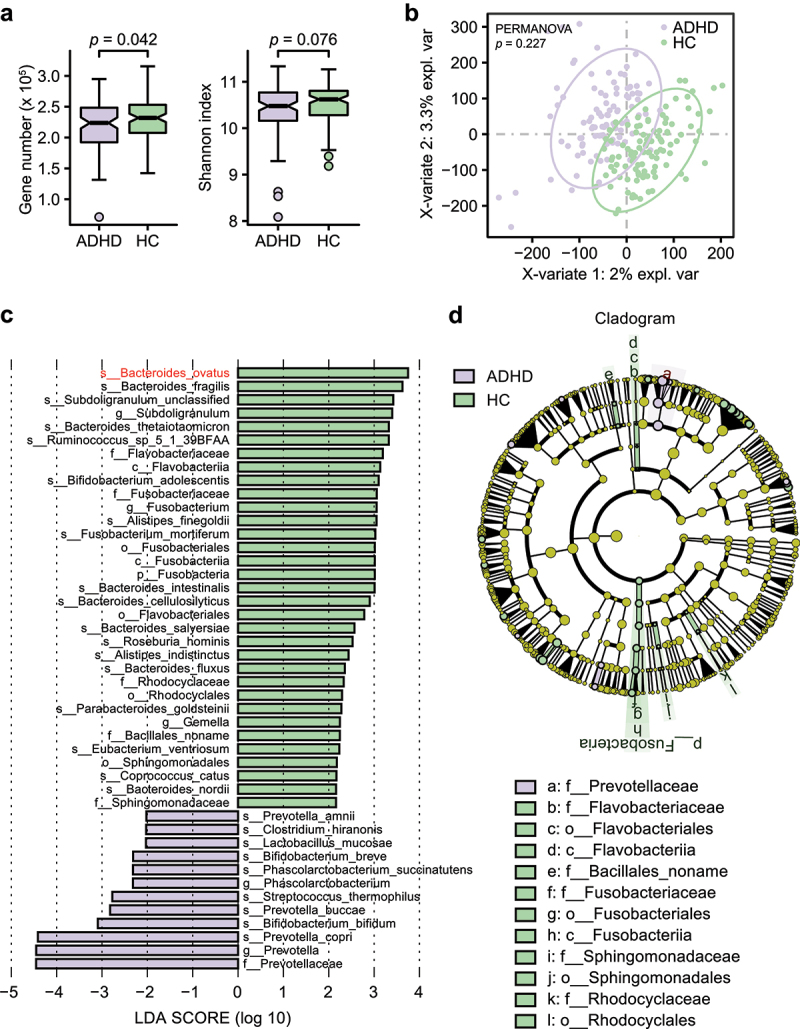
**Alpha and beta diversity analyses in all patients with ADHD and HCs**. (a) Gene count and alpha diversity (Shannon index) in all patients with ADHD and HCs. The Wilcoxon rank-sum test was used to determine significance. (b) Supervised analysis with sparse PLS-DA in all patients with ADHD at the gene level. PERMANOVA calculation based on the Bray–Curtis distance at the gene level. (c) LDA effect size analysis identified significantly different taxa between the total ADHD cohort and HCs. The LDA scores (log 10) > 2 and *p* < .05 are shown. A negative LDA score indicated enrichment in patients with ADHD (purple), while a positive LDA score indicated enrichment in HCs (green). Bar length indicates the effect size of each taxon. (d) Taxonomic cladogram obtained from LEfSe analysis. The circles from inside to outside represent different classification levels, and the size of each dot is proportional to its relative abundance. The colored taxa represent significantly different taxa between the total ADHD patient cohort and HCs. Purple, total ADHD-enriched; Green, HC-enriched.

The differences in the alpha diversity of the gut microbiota between the total ADHD patient cohort and HCs was estimated. We found significantly lower gene numbers in patients than in HCs (*p*= .042), although the gut microbiota richness was similar between the two groups (Shannon index, *p* = .076, Figure 1a). To assess whether the gut microbiota could be effectively distinguished between the two groups, supervised analysis with sparse PLS-DA (sPLS-DA) and permutational multivariate analysis of variance (PERMANOVA) were performed at the gene family level. In the sPLS-DA results, ADHD and HC samples clustered into two groups, although the PERMANOVA results suggested that the microbial composition was not significantly different between the two groups (*p* = .227, Figure 1b).

Metagenomic sequencing analysis (LEfSe) identified 12 bacterial taxa that were enriched in patients and 33 that were enriched in HCs (Figure 1c). At the species level, eight of the top 18 HC-enriched species (*ovatus, fragilis, thetaiotaomicron, intestinalis, cellulosilyticus, salyersiae, fluxus*, and *nordii*) belonged to the genus *Bacteroides*. Species in *Bifidobacterium* (*breve* and *bifidum*) and *Prevotella* (*amnii, buccae* and *copri*) were more abundant in patients with ADHD than in HCs. A cladogram of significantly different taxa is shown in Figure 1d, and an overview of the relative abundance of different bacteria at the genus level between patients and HCs is shown in Table S4.

Moreover, we found a small number of viruses and eukaryotes in some individuals, but archaea were not detected in any individuals. There were no significant differences between the amounts of viruses and eukaryotes (Table S5).

#### Fecal microbiome diversity in ADHD patient subgroups

**Figure 2.**
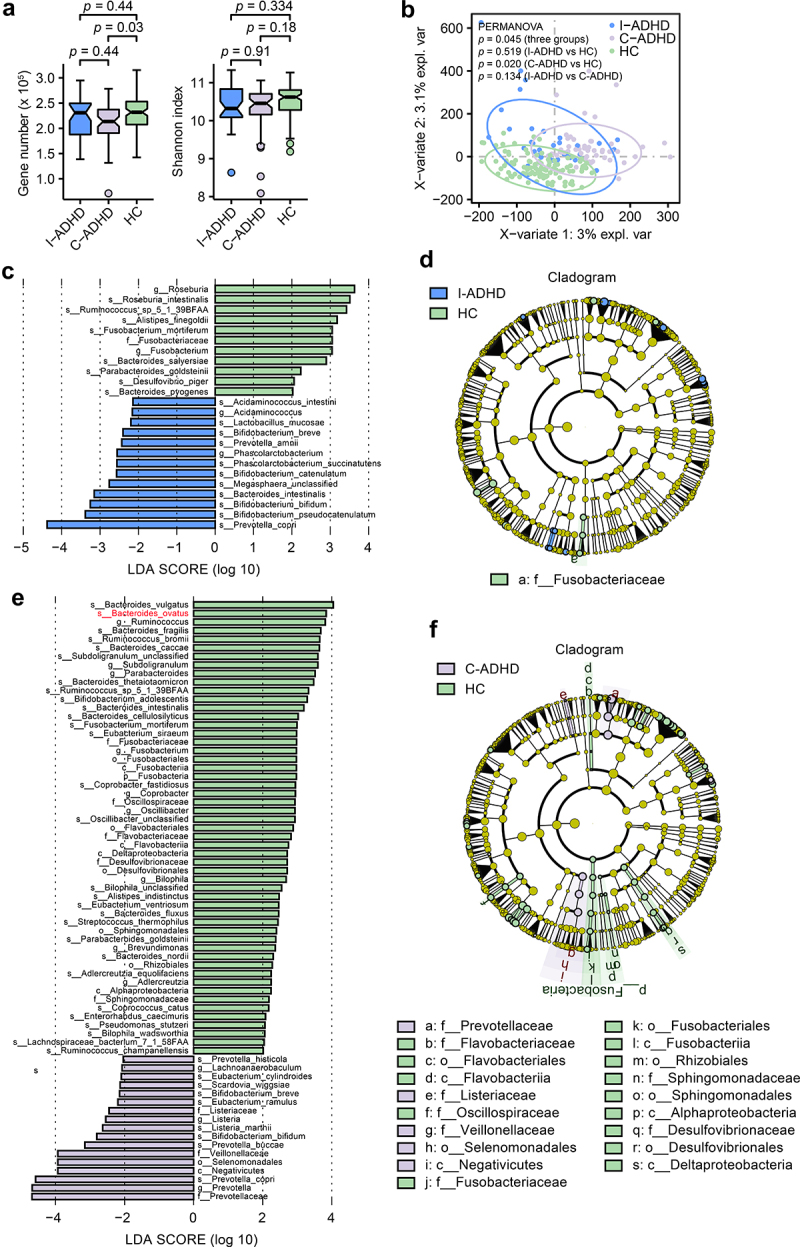
**Alpha and beta diversity analyses in subgroups of patients with ADHD and HCs**. (a) Gene count and alpha diversity (Shannon index) in patient subgroups and HCs. The Wilcoxon rank-sum test with Benjamini Hochberg adjustment was used to determine significance. (b) Supervised analysis with sparse PLS-DA in patient subgroups at the gene level. PERMANOVA calculation based on the Bray–Curtis distance at the gene level. LDA effect size analysis identified significantly different taxa between I-ADHD and HC (c) and between C-ADHD and HC (e). The LDA score (log 10) > 2 and *p* < .05 are shown. Bar length indicates the effect size of each species. Taxonomic cladogram obtained from LEfSe analysis for different comparisons (D, I-ADHD vs. HC; F, C-ADHD vs. HC). The circles from inside to outside represent different classification levels, and the size of each dot is proportional to its relative abundance. The colored taxa represent significantly different taxa between the ADHD and HC subgroups. Blue, I-ADHD-enriched; purple, C-ADHD-enriched; Green, HC-enriched.

The analysis of gene numbers in patient subgroups indicated that there was lower dissimilarity between patients with I-ADHD and HCs (*p* = .44) than between patients with C-ADHD and HCs (*p* = .03, Figure 2a). However, alpha diversity analysis (Shannon index) revealed no significant difference between any two groups among C-ADHD, I-ADHD and HC. The sPLS-DA results showed a significant dissimilarity between patients with C-ADHD and HCs (*p* = .020, Figure 2b). No significant findings were obtained between patients with I-ADHD and HCs (*p* = .134) or between patients with I-ADHD and C-ADHD (*p* = .519).

Metagenomic sequencing analysis (LEfSe) identified 13 bacterial taxa that were enriched in patients with I-ADHD and 11 that were enriched in HCs when the two groups were contrasted (Figure 2c). In addition, seventeen bacterial taxa were enriched in patients with C-ADHD, and 53 were enriched in HCs (Figure 2e) in comparisons between the two groups. Cladograms of the taxa that were significantly different between the ADHD subgroup and HCs are shown in Figures 2d and 2f, respectively. An overview of the relative abundances of different bacteria at the genus level between ADHD subgroup patients is shown in Table S6. Together, these results suggest greater variation between the gut microbiota of C-ADHD patients and HCs than between those of I-ADHD patients and HCs.

Of note, there was a higher proportion of male subjects in the C-ADHD subgroup than in HCs. To address whether the differences in the gut microbiota exhibited in Figure 1b were associated with the sex differences between the two groups, we further performed analyses stratified for males. The gene numbers (*p* = .057) and gut microbiota richness (Shannon index, *p* = .076) in males were similar between the total ADHD patient cohort and HCs (Fig. S1A). The results of the comparison of the gene numbers (*p* = .05) and gut microbiota richness (Shannon index, *p* = .23) in the male subjects of the C-ADHD and HCs were similar to the results obtained in the analyses performed with both sexes (Fig. S1B). PERMANOVA results showed a more significant difference in male samples between C-ADHD and HCs (*p* < .001, Fig. S1D) than between the total ADHD cohort and HCs (*p* = .028, Fig. S1C). These data suggest that unmatched sex did not alter the outcome of the analysis, in which greater variation in the gut microbiota between C-ADHD patients and HCs was demonstrated.

**Figure 3.**
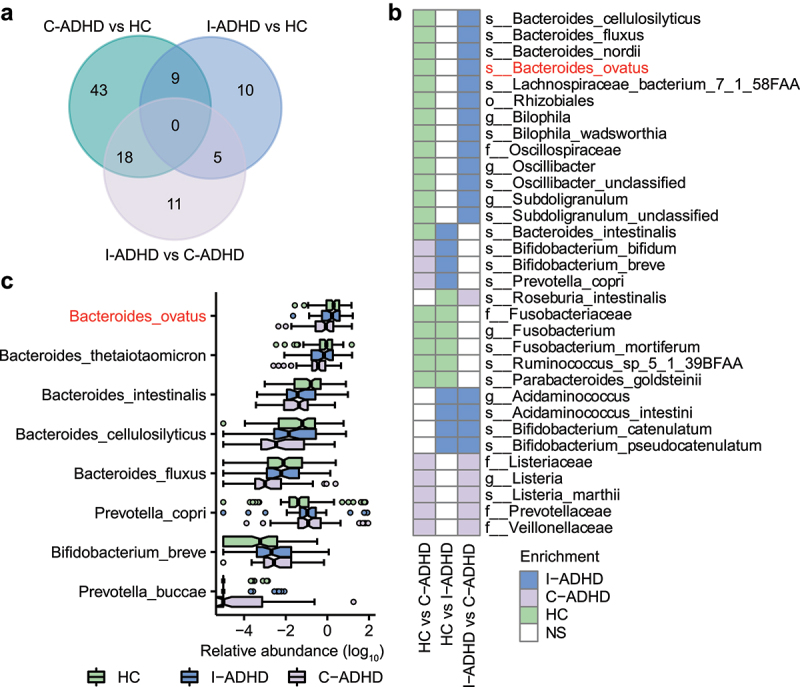
**The gut microbial composition of C-ADHD is different from that of I-ADHD and HCs**. (a) Venn diagram of significantly different taxa among different comparisons. (b) Heatmap of shared significantly different taxa among different comparisons. LEfSe analysis was used to detect significantly different taxa in (A) and (B). (c) Boxplot of significantly different species among HCs, I-ADHD, and C-ADHD. Significant differences among groups were evaluated using the Jonckheere-Terpstra test and adjusted by the Benjamini–Hochberg (BH) method (*p* < .05).

We further compared the numbers of significantly different bacterial taxa among the three groups: C-ADHD vs. HCs (70), I-ADHD vs. C-ADHD (34), and I-ADHD vs. HCs (24) (Figure 3a). The patterns of bacterial differences between C-ADHD and HCs and between C-ADHD and I-ADHD were largely shared (Figures 3a, b). Thirteen gut bacterial taxa that were more abundant in HCs than in patients with C-ADHD were also enriched in patients with I-ADHD. The bacterial taxa that were enriched in both the HCs and the I-ADHD subgroup were *Rhizobiales, Oscillospiraceae, Bilophila, Oscillibacter, Subdoligranulum, Bacteroides cellulosilyticus, Bacteroides fluxus, Bacteroides nordii, Bacteroides ovatus, Lachnospiraceae bacterium, Bilophila wadsworthia, Oscillibacter unclassified*, and *Subdoligranulum unclassified*. The other 5 bacterial taxa (*Listeriaceae, Prevotellaceae, Veillonellaceae, Listeria*, and *Listeria marthii*) were more abundant in patients with C-ADHD than in patients with I-ADHD or HCs. Together, these data provide preliminary evidence of greater dissimilarity in the gut microbial composition between patients with C-ADHD and HCs than between patients with I-ADHD and HCs. Notably, patients with C-ADHD can be distinguished from patients with I-ADHD by different gut microbiota profiles.

In addition, the Jonckheere-Terpstra test identified a progressive prevalence or scarcity from HCs to patients with I-ADHD and C-ADHD (Figure 3c). We found that *Prevotella copri, Prevotella buccae* and *Bifidobacterium breve* were progressively enriched from HCs to patients with I-ADHD and C-ADHD, while progressively reduced enrichment of the species *ovatus, thetaiotaomicron, intestinalis, cellulosilyticus*, and *fluxus* belonging to the genus *Bacteroides* was also identified. These results suggest that the progressively increased or reduced enrichment of microbial taxa may be associated with the different ADHD symptomatic profiles.

#### Gut microbial taxa associated with ADHD clinical characteristics

**Figure 4.**
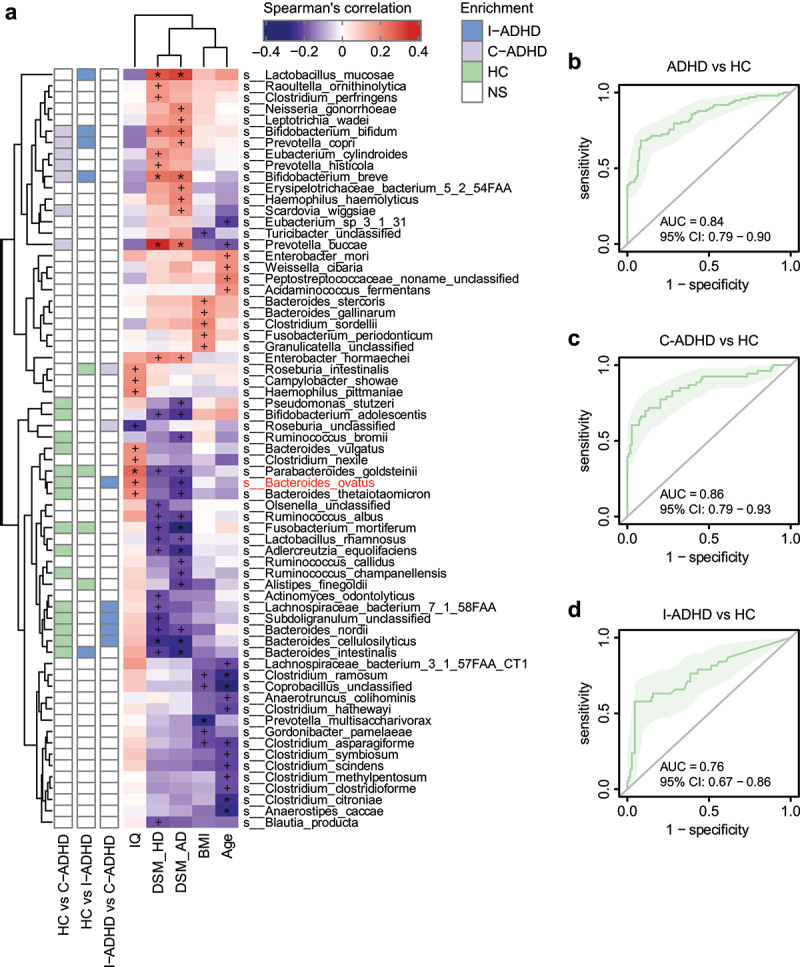
**Correlation between gut microbiota species and ADHD clinical characteristics**. (a) Heatmap of Spearman’s correlation coefficient between the relative abundance of species and ADHD clinical characteristics (red and purple for positive and negative correlation, respectively). The species enrichment direction in different comparisons is shown on the left. Blue, I-ADHD-enriched; purple, C-ADHD-enriched; green, HC-enriched; gray, no significant difference. ‘+’ denotes *p* < .01; ‘*’ denotes *p* < .001. Classification of samples among different groups by relative abundance at the species level (b-d). (b) ROC generated between patients with ADHD and HCs by 6 microbial markers selected by the random forest model. The AUC was 0.84, and the 95% CI was 0.79–0.90 (green area). (c) ROC generated between patients with C-ADHD and HCs by 8 microbial markers selected from the random forest model. The AUC was 0.89, and the 95% CI was 0.84–0.95. (d) ROC generated between patients with I-ADHD and HCs by 35 microbial markers selected by the random forest model. The AUC was 0.76, and the 95% CI was 0.67–0.86.

The associations between the composition of the gut microbiota and clinical symptoms in ADHD were also assessed. Spearman’s rank correlation analyses showed significant correlations of bacterial species with symptom severity scores (Figure 4a). The species that were more abundant in patients with C-ADHD than in HCs, which included *Prevotella buccae, Bifidobacterium breve*, and *Bifidobacterium bifidum*, were enriched and positively associated with both DSM_HD and DSM_AD scores. Several species belonging to the genus *Bacteroides* that were abundant in HCs were negatively correlated with DSM_HD and/or DSM_AD scores. We found that increased relative abundances of *Bacteroides nordii, Bacteroides cellulosilyticus* and *Bacteroides intestinalis* were associated with fewer symptoms of both hyperactivity/impulsivity (DSM_HD scores) and inattention (DSM_AD scores). *Bacteroides thetaiotaomicron* and *Bacteroides ovatus* were negatively associated only with DSM_AD scores. Moreover, we repeated this analysis, excluding the healthy controls, to verify that the results of these clinical indices used to assess the correlation of the microbiome with clinical features would persist. The clinical index used to perform microbiome analysis in patients with ADHD suggested that a high abundance of *Bacteroides* was associated with reduced DSM scores. Alternatively, a high relative abundance of *Prevotella* correlated with increased DSM scores (Fig. S2). These findings were very similar to the results of the initial analysis that included all groups, suggesting the significant role that *Bacteroides* and *Prevotella* play in ADHD.

Random forest (RF) classifications were established based on these differentiated gut bacterial species. The relative abundances of 6 bacterial species (Fig. S3A) distinguished the total ADHD patient cohort from the HCs (Figure 4b; AUC = 0.84, 95% CI 0.79–0.90). For ADHD subgroup classifications, 9 selected species (Fig. S3B) distinguished patients with C-ADHD from HCs (Figure 4c) with an AUC of 0.86 (95% CI 0.79–0.93), while the relative abundances of 3 bacterial species (Fig. S3C) distinguished patients with I-ADHD from HCs (Figure 4d; AUC = 0.76, 95% CI 0.67–0.86). These results indicate that these classifiers were able to differentiate patients with C-ADHD from HCs with good performance.

#### Distinct bacterial functional profiles in ADHD subgroups

Microbial metagenomic sequencing data were used to predict the discrepancies in the functional metabolic pathways in the total ADHD patient cohort and in patient subgroups. Altogether, 362 pathways from the MetaCyc metabolic pathway database that were present in more than 10% of samples were identified and analyzed. A total of 9 pathways were significantly different between the total ADHD patient cohort and HCs (*p* < .05, LDA score > 2; Fig. S4A). LEfSe results revealed that only bacterial xylose degradation IV was significantly enriched in patients with ADHD, while the activation of bacterial pathways for inosine-5’-phosphate biosynthesis II, flavin biosynthesis III (fungi), L-phenylalanine degradation IV, adenine and adenosine salvage III, starch degradation V, hydrogen production VIII, purine ribonucleoside degradation and L-rhamnose degradation I was significantly reduced in patients with ADHD.

Subsequently, the diversity of the functional pathways in the ADHD patient subgroups was assessed. The analysis predicted that the C-ADHD subgroup had higher activation of bacterial pathways for S-adenosyl-L-methionine cycle I and xylose degradation and lower activation of GDP-mannose-derived O-antigen building block biosynthesis, pantothenate and coenzyme A biosynthesis III, L-histidine biosynthesis, L-arginine biosynthesis III, L-rhamnose degradation I, flavin biosynthesis III, inosine-5’-phosphate biosynthesis I, pyridoxal 5’-phosphate biosynthesis and salvage and NAD salvage pathway I than those of the HCs (Fig. S4B). The analysis also predicted that the I-ADHD subgroup exhibited higher activation of bacterial pathways of L-lysine, L-threonine and L-methionine biosynthesis I, anhydromuropeptide recycling, tetrahydrofolate biosynthesis and salvage and heme biosynthesis from glycine, and lower activation of pathways of pyrimidine ribonucleotide de novo biosynthesis, purine ribonucleoside degradation, GDP-mannose biosynthesis and stachyose degradation than those in the HCs. Of note, the Venn diagram illustrates that there is zero overlap in the different pathway profiles observed in C-ADHD vs. HCs and I-ADHD vs. HCs (Fig. S4C).

### Animal studies

#### *Bacteroides ovatus* ATCC8483 supplementation corrected spatial working memory impairment in ADHD rats

Early life stage event-associated environmental factors may account for the diverse composition of the gut microbiota and the development of many psychiatric disorders via the microbiota-gut-brain axis.^16^ Previous studies have shown that SHRs exhibit the core symptoms of ADHD, including but not limited to hyperactivity, attention deficits, and impulsivity.^41^ Here, we found that the abundance of *Bacteroides ovatus* was remarkably lower in patients with ADHD or C-ADHD than in HCs. *Bacteroides ovatus* was identified as the species that principally promoted the production of gut secretory IgA and maintained gut homeostasis.^42^ *Bacteroides ovatus* was also found to consume gut tryptophan and produce indole-3-acetic acid, an important tryptophan metabolite,^43^ which drives hippocampal neurogenesis in adult mice.^44^ Therefore, we next wondered whether exogenous *Bacteroides ovatus* supplementation could affect brain function and ADHD-like behaviors in SHRs.

**Figure 5.**
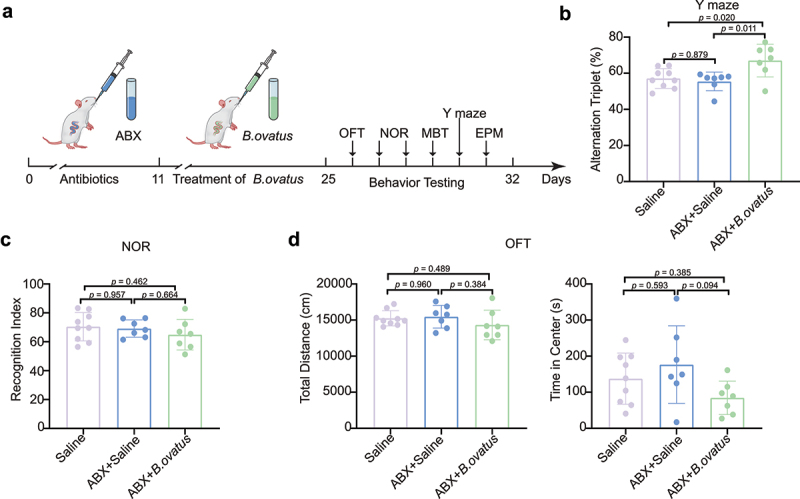
***Bacteroides ovatus* ATCC8483 gavage ameliorated spatial working memory in SHRs**. (a) Schematic diagram of *Bacteroides ovatus* transplantation and behavioral tests. There were three groups (per group = 7–9 rats): Saline (gavage with saline during the first 24 days as a control), ABX+Saline (treated with an antibiotic cocktail within the first 10 days and saline for the next 14 days via oral gavage and drinking water), and ABX+*B. ovatus* (treated with an antibiotic cocktail within the first 10 days and *Bacteroides ovatus* for the next 14 days via oral gavage and drinking water). (b) Spontaneous alternations of each group were recorded in the Y maze. (c) Comparison of the recognition index among different rat groups. (d) For the open field test, the time spent in the center and the total distance of locomotion of each group were analyzed. Mean ± SEM are plotted; one-way ANOVA followed by the Tukey–Kramer post hoc test. ABX: antibiotic cocktail, OFT: open field test, NOR: novel object recognition, MBT: marble burying test, EPM: elevated plus maze.

Four-week-old SHRs were exposed to *Bacteroides ovatus* by oral gavage after administration of broad-spectrum antibiotic cocktail (ABX) (Figure 5a). We found that the obligate anaerobic *B. ovatus* was still viable in the drinking water for at least 6 h (Fig. S5). The ABX+*B. ovatus*-treated and ABX*+Saline*-treated SHRs showed restored Shannon diversity (Fig. S6A) and microbiome structure (Fig. S6B) unlike the saline-treated SHRs. The abundance of gut *Bacteroides ovatus* in SHRs was measured by amplifying its 16S rRNA gene (Fig. S6C). We found that *Bacteroides ovatus* treatment markedly rescued the spatial working memory and inattention of SHRs in the Y maze test unlike with saline treatment (*p* = .020, Figure 5b). However, novel object preference was indistinguishable between *Bacteroides ovatus*-treated and saline-treated SHRs (Figure 5c). Notably, locomotor activities (Figure 5d) and anxiety-like behaviors, including those assessed by the number of buried marbles and time spent in the open arms of the elevated plus maze (Fig. S7), were not changed by supplementation with *Bacteroides ovatus*. Moreover, we found that *Escherichia coli* transplantation did not alleviate the impaired spatial working memory and other ADHD-like behaviors in SHRs (Fig. S8). Together, these results suggest that *Bacteroides ovatus* specifically modulates spatial working memory instead of recognition memory and that memory improvement is not dependent on altered locomotion or anxiety.

#### *Bacteroides ovatus* ATCC8483 treatment reversed the enhanced θ EEG rhythms of ADHD rats

**Figure 6.**
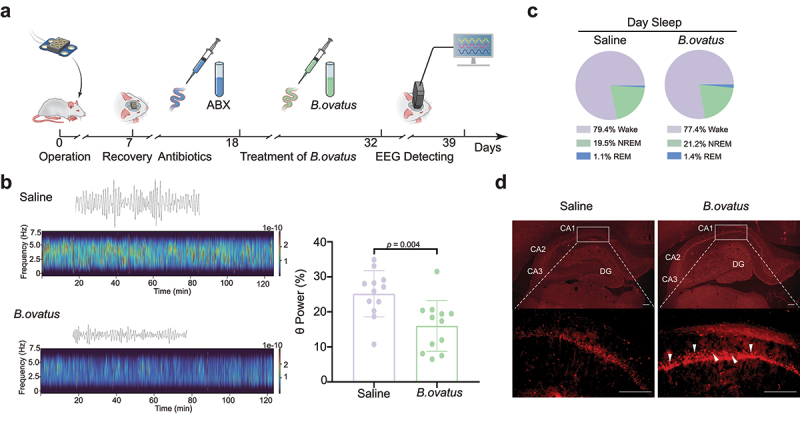
***Bacteroides ovatus* ATCC8483 administration corrected aberrant θ EEG rhythms and activated hippocampal neurons in SHRs**. (a) Schematic diagram of *Bacteroides ovatus* transplantation and EEG recording. There were two groups (per group = 12 rats): Saline (treated with an antibiotic cocktail within the first 10 days and saline for the next 14 days via oral gavage and drinking water as a control) and *B. ovatus* (treated with an antibiotic cocktail within the first 10 days and *Bacteroides ovatus* for the next 14 days via oral gavage and drinking water). (b) Representative wave and spectrogram of θ EEG rhythms in saline and *B. ovatus*-colonized SHRs and θ rhythm percentage in the total EEG. (c) The proportion of REM sleep, NREM sleep, and wakefulness in saline- and *B. ovatus*-treated SHRs, recorded by EEG/EMG. (d) cFos^+^ neurons in the hippocampal CA1, CA2, CA3, and DG subregions of SHRs 90 min after the Y maze test at day 39. Scale bar = 200 μm. Mean ± SEM are plotted; 2-tailed Student’s *t* test. REM: rapid eye movement, NREM: nonrapid eye movement, DG: dentate gyrus.

We next wondered about the possible mechanisms underlying the above *Bacteroides ovatus*-mediated amelioration of spatial working memory in SHRs. EEG rhythms, representing gross brain activity, were recorded in SHRs after administration of *Bacteroides ovatus* (Figure 6a). It has been repeatedly reported that children with ADHD and other ADHD animal models, including SHRs, exhibit an increased proportion of θ-band power EEG rhythms.^45,46^ Here, we found that *Bacteroides ovatus*-treated SHRs showed significantly lower θ EEG rhythms than those of saline-treated SHRs (*p* = .004, Figure 6b), without alteration of the proportion of sleep phases (Figure 6c). Collectively, these EEG data suggest that the enhancement in spatial working memory induced by *Bacteroides ovatus* is related to improved brain function.

#### *Bacteroides ovatus* ATCC8483 triggered hippocampal neuron activation

The formation of spatial working memory requires interaction across the hippocampus and its adjacent cortices.^47^ To identify *Bacteroides ovatus*-associated brain activation patterns, we repeated the Y maze test on day 39 after EEG recording. The expression of c-Fos and the representation of neuronal activation were investigated 90 min after the Y maze test. We found that c-Fos espression was mainly increased in the hippocampal CA1 subregion but not in the CA2, CA3 or dentate gyrus (DG) subregions after *Bacteroides ovatus* treatment (Figure 6d). Moreover, c-Fos expression was not changed in other regions known to be associated with ADHD, including the medial prefrontal cortex (mPFC) and caudate-putamen (CPU; a.k.a. dorsal striatum) (Fig. S9). Together, we identified neuronal activation in the hippocampal CA1 subregion, suggesting that the CA1 subregion is responsible for *Bacteroides ovatus*-induced spatial working memory enhancement in SHRs.

## Discussion

In the present study, we characterized the metagenomic profiles of the gut bacterial population in ADHD generally and in its two major symptomatic classifications. Several microbial taxa were identified to be associated with the clinical parameters and severity of ADHD, including the C-ADHD-depleted bacterial species, *Bacteroides ovatus*. We confirmed this finding by rescuing spatial working memory, EEG rhythms and hippocampal neuronal activation in *Bacteroides ovatus*-transferred ADHD rats. These results not only identify alterations in the composition of the gut microbiome in ADHD patient subgroups but also, more importantly, provide a potential bacteria-based therapeutic strategy to be tested for their use in treating certain deficits associated with ADHD.

The metagenomic data demonstrated gut dysbiosis in patients with ADHD unlike in HCs, which is consistent with the previous results of 16S rRNA gene amplicon sequencing analysis.^21–26^ We found a significantly lower gene number in patients with C-ADHD than in HCs, although the gut microbiota richness, namely, the alpha diversity, was similar between any two groups. There were also no significant differences observed in alpha diversity between patients with ADHD and HCs in some previous studies.^21,22,26,48^ Regarding beta diversity, the current study revealed that the gut microbiota in patients with C-ADHD can be effectively distinguished from that of HCs, but those of patients with I-ADHD and HCs cannot be distinguished. Of note, these results indicated that the analysis of the comparison between patients with C-ADHD and HCs could reveal more significant differences in bacterial diversity, which may be missed when all patients with ADHD are compared with HCs.

Notably, the bacterial taxa that are reported to be distinct between patients with ADHD and HCs were highly inconsistent in previous studies.^21–26,48^ The results indicating that certain bacterial taxa were altered in one cohort may never be replicated, or opposite trends in abundance may be observed in another cohort. This discrepancy might reflect differences in the size of the cohort and the age, sex, region, diet, medication use, early life environment, maternal health, and cesarean delivery status of the subjects, since all these factors could affect gut microbial composition. Here, we found underrepresentation of 8 species (*ovatus, fragilis, thetaiotaomicron, intestinalis, cellulosilyticus, salyersiae, fluxus*, and *nordii*) belonging to the genus *Bacteroides* in the total ADHD patient cohort. Members of the genus *Bacteroides* are usually beneficial for gut function and are correlated with neurodevelopment.^49,50^ In addition, species in *Bifidobacterium* (*breve* and *bifidum*) and *Prevotella* (*amnii, buccae* and *copri*) were more abundant in the total ADHD patient cohort than in the HCs. Although we did not find considerable overlap with the previously reported microbial signature of ADHD, the increased abundance of *Bifidobacterium breve* and *Bifidobacterium bifidum* agreed with the results of a Dutch cohort study.^21^

Regarding the ADHD subgroups, the number of bacteria taxa that were different between C-ADHD and HC was the largest, followed by that between I-ADHD and C-ADHD and that between I-ADHD and HC. We found a slight difference in the bacterial populations between I-ADHD patients and HCs, and we speculate that the relatively minor symptoms of I-ADHD may be involved in this difference. Thus, we identified several progressively enriched microbial taxa from HCs to patients with I-ADHD and C-ADHD, which is consistent with the clinical severity of ADHD.^4,51^ Interestingly, the enrichment of the species *Prevotella copri, Prevotella buccae* and *Bifidobacterium breve* progressively increased, while that of *ovatus, thetaiotaomicron, intestinalis, cellulosilyticus* and *fluxus* belonging to the genus *Bacteroides* progressively decreased from HCs to patients with I-ADHD and C-ADHD. Moreover, *Prevotella, Bifidobacterium* and *Bacteroides* were also associated with hyperactivity/impulsivity and/or inattention symptoms. Taken together, these findings might indicate that there were distinguishable gut microbial patterns in the I-ADHD and C-ADHD subgroups. We can obtain more accurate information on the gut microbiota from subgroup analysis, which is potentially helpful in the diagnosis of ADHD subtypes. Notably, however, an increased abundance of *Bacteroides ovatus* was found in a general ADHD patient cohort in a previous small-sized study.^24^ The proportion of patients with C-ADHD in the general patient cohort may be involved in the discrepancy in *Bacteroides ovatus* abundance observed between the present study and the previous one.

Some studies have analyzed the association between dietary habits and ADHD. The common finding is that unhealthy dietary patterns (i.e., high in saturated fat and refined sugars and low in fruit and vegetables) are associated with ADHD.^52,53^ Here, the dietary patterns of participants do not significantly affect the composition of gut microbiota. However, the recording used to assess the food classes consumed by the patients in the present study was at a very rough level, which indicates a dietary preference for vegetables or whole grains.

The functional metabolic pathways predicted from fecal metagenomic analysis exhibited additional divergence in patients with ADHD. We found that the activation of functions correlated with energy regulation in host metabolism, including inosine-5’-phosphate biosynthesis, flavin biosynthesis, adenine and adenosine salvage and purine ribonucleoside degradation, was reduced in ADHD patients. However, it is unclear whether these alterations contribute to abnormal host symptoms, although aberrant brain metabolism is involved in some psychiatric disorders.^54^ The starch degradation, hydrogen production and rhamnose degradation pathways were also predicted to exhibit decreased activation in patients, and these metabolic pathways were associated with host gut functions.^55–57^ Moreover, relatively enriched xylose degradation and reduced phenylalanine degradation pathways in patients with ADHD are particularly striking. The disturbance of xylose metabolism is implicated in *Drosophila* hyperactivity behavior.^58^ Phenylalanine is the precursor of dopamine, and the latter is well studied and known as a dominant neurotransmitter that is deficient in ADHD pathophysiology.^59,60^ Although very few functional analyses predicted bacterial profiles in previous studies, the increased levels of cyclohexadienyl dehydratase, responsible for phenylalanine synthesis, predicted in the study of the Dutch cohort^21^ and the current data together suggest a critical role of abnormal phenylalanine metabolism in patients with ADHD. Moreover, the genus *Bifidobacterium* has been reported to affect the level of cyclohexadienyl dehydratase.^21^ Microbial products and metabolites can signal through enteroendocrine cells and enterochromaffin cells to modulate the secretion of neuropeptides, neuromodulators, and neurotransmitters.^61^ Therefore, insufficient dopamine signals in the brain may induce potentially compensated precursor production through the microbiota-gut-brain axis.

The functional analysis performed in subgroups showed some shared and distinct differential metabolic pathways in patients with C-ADHD and the total patient cohort. Notably, the activation of arginine and pyridoxal 5’-phosphate biosynthesis pathways were specifically reduced in the C-ADHD subgroup. Arginine, a precursor of nitric oxide, is related to better memory and improved intestinal inflammation.^62,63^ Pyridoxal 5’-phosphate is a dominant vitamin B6 active type, and serum vitamin B6 levels were found to be decreased in ADHD patients.^64^ Moreover, there was no overlap in the pathways that were different between C-ADHD and HCs and between I-ADHD and HCs, suggesting distinct gut bacterial functions in ADHD subgroups.

Although additional microbial sequencing data have been reported to discriminate gut bacterial alterations in patients with ADHD,^65–67^ the evidence from mechanistically supporting experiments to confirm the proposed link was weak, especially considering the results of host-based bacterial transplantation experiments. Interestingly, anxiety-like behavior was elicited in mice colonized with the fecal microbiota of patients with ADHD.^14^ Here, we identified 33 total ADHD-depleted and 53 C-ADHD-depleted gut bacteria. We focused on these microbes with decreased abundances and aimed to restore their abundances in ADHD rats. The abundance of *Bacteroides ovatus* was significantly lower in patients with ADHD or C-ADHD than in HCs. *Bacteroides ovatus* was identified as the species that produces gut secretory IgA and limits mucosal inflammation.^42^ *Bacteroides ovatus* was also found to consume gut tryptophan and produce indole-3-acetic acid, and the latter drives adult hippocampal neurogenesis in mice.^43,44^ Therefore, *Bacteroides ovatus* colonization may benefit the regulation of circulatory inflammation and cerebral function, although this requires further experiments to assess. *Bacteroides ovatus*-monocolonized ADHD rats exhibited significantly increased spontaneous alternations in the Y maze task, indicating an improvement in spatial working memory and inattention. Of note, the abundance of *Bacteroides ovatus* was negatively associated with DSM_AD scores but not DSM_HD scores, which may underlie the unaltered locomotor activity in ADHD rats receiving *Bacteroides ovatus* colonization. Moreover, *Bacteroides ovatus* monotherapy was found to be superior to traditional fecal transplantation and multistrain bacteriotherapy (triple Bacteroides: *B. ovatus, B. thetaiotaomicron*, and *B. vulgatus*) in a murine colitis model.^68^

Here, we also found that *Bacteroides ovatus* colonization can normalize EEG rhythms and specifically activate hippocampal neurons in ADHD rats. Interestingly, the hippocampal volumes in patients with ADHD were observed to be lower than those in HCs, which has been repeatedly reported by brain image analysis;^69,70^ this finding suggests that *Bacteroides ovatus* supplementation contributed to ameliorating hippocampal function and hippocampus-associated spatial working memory in these ADHD rats.

In addition, we found underrepresentation of 8 species (*ovatus, fragilis, thetaiotaomicron, intestinalis, cellulosilyticus, salyersiae, fluxus*, and *nordii*) belonging to the genus *Bacteroides* in patients with ADHD. The different cocktail treatments incorporating these *Bacteroides spp*. may produce different effects on ADHD rats, which deserves further study.

The main strengths of the present study include the use of metagenomic sequencing, subgroup analyses and a larger sample size than those in previous studies. Second, only medication-naive patients with ADHD were recruited in the current study to exclude the effects of medication on the gut microbiota. Additionally, patients with psychiatric or gastrointestinal comorbidities were also excluded. Given that the gut microbiome composition is highly correlated with dietary habits, a questionnaire assessing general diet and defecation habits was collected from individual participants to assess dietary differences between groups. Third, to our knowledge, this is the first study using single bacterial species transplantation to link the gut-brain axis mechanism in ADHD. There are also some limitations of this study. First, distinct gut profile information for patients with HI-ADHD could not be obtained in our study due to an insufficient number of patients. Second, in line with a real-world scenario, the groups were not completely matched by sample size, sex, only child status, percentage of cesarean section and low birth weight, although we found that unmatched sex did not alter the outcome of the greater variation in gut microbiota observed between C-ADHD and HCs. Third, we performed a cross-sectional study, and further longitudinal work should be conducted to further assess age- or medication-associated variations in gut microbiota. Finally, the specific molecular and metabolic mechanisms underlying gut *Bacteroides ovatus*-mediated improvements in brain function deserve further study.

In conclusion, our study characterized the distinct gut microbiota panel in patients with ADHD and its subgroups. We found more gut microbial alterations in patients with C-ADHD than in patients with I-ADHD. We also identified several progressively enriched or decreased microbial taxa from HC to I-ADHD and C-ADHD, which is consistent with the clinical severity of ADHD. *Bacteroides ovatus* transplantation in ADHD rats rescued spatial working memory and attention-associated recognition function. The current study provides new evidence supporting that the microbiota-gut-brain axis is associated with ADHD.^71^

## Supplementary Material

Supplemental Material

## Data Availability

The metagenomic shotgun sequencing data for all samples have been deposited in the CNGB Nucleotide Sequence Archive (CNSA) under accession code CNP0000729. Other data that support the findings of this study are available within the paper and its supplementary files or from the corresponding author upon reasonable request.
