# Supplementary material for: Gut metagenomic characteristics of ADHD reveal low Bacteroides ovatus-associated host cognitive impairment
Source: Gut Microbes. 2022 Sep 20;14(1):2125747. doi: 10.1080/19490976.2022.2125747 (PMC9519028; doi:10.1080/19490976.2022.2125747)
Supplement: Supplemental Material [file KGMI_A_2125747_SM1185.zip › 3 Supplemental material 1 20220816.docx]

**Gut metagenomic characteristics of ADHD reveal low *Bacteroides ovatus*-associated host cognitive impairment**

*Yan Li, Haiting Sun, Yufen Huang, Anqi Yin, Linjuan Zhang, Jiao Han, Yixuan Lyu, Xiangzhao Xu, Yifang Zhai, Huan Sun, Ping Wang, Jinyang Zhao, Silong Sun, Hailong Dong, Feng zhu, Qiang Wang, Luis Augusto Rohde, Xuefeng Xie, Xin Sun, and Lize Xiong*

**Supplementary Materials Index**

**Supplementary Figures 1–9**

Fig. S1 Alpha and beta diversity analyses in male patients with ADHD and HCs.

Fig. S2 Correlation between gut microbiota species and clinical characteristics of ADHD patients.

Fig. S3 Selection of microbial markers used to discriminate the total patients with ADHD and HCs. (A), C-ADHD and HCs (B), I-ADHD and HCs (C) based on a random forest model.

Fig. S4 Gut microbial functions of C-ADHD are different from those of I-ADHD and HCs.

Fig. S5 Verification of *Bacteroides ovatus* viability in drinking water.

Fig. S6 The gut microbiota changes in SHRs 14 days after bacterial transplantation.

Fig. S7 Anxiety behaviors were not altered in SHRs after *Bacteroides ovatus* ATCC 8483 gavage.

Fig. S8 ADHD-like behaviors were not altered in SHRs after *Escherichia coli* AM12-30 gavage.

Fig. S9 Administration of *Bacteroides ovatus* ATCC 8483 did not activate the neurons in the CPU or mPFC of SHRs.

**Supplementary Tables 1-6**

Table S1 Influence of host clinical characteristics on gut microbiota.

Table S2 Dietary habits of the study cohort.

Table S3 Defecation characteristics of the study cohort.

Table S4 Relative abundances of different bacterial genera in the total ADHD patient cohort and HCs.

Table S5 Relative abundances of eukaryota and viruses between ADHD and HCs.

Table S6 Relative abundances of different bacterial genera among I-ADHD, C-ADHD and HCs.

**Supplementary Figures**


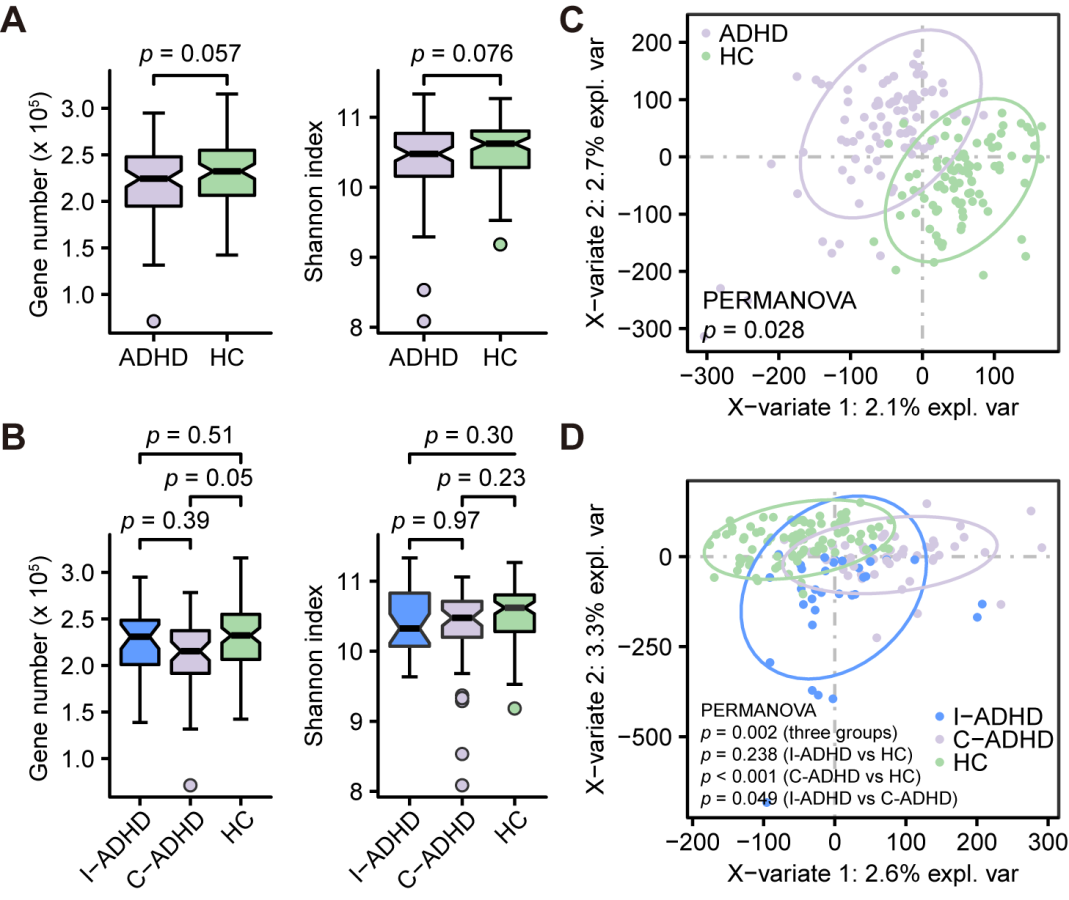


**Fig. S1 Alpha and beta diversity analyses in male patients with ADHD and HCs**. (A, B) Gene count and alpha diversity (Shannon index) in ADHD (A, n = 90) or subgroup patients (B, I-ADHD, n = 33; C-ADHD, n = 52) and HCs (n = 89). The Wilcoxon rank-sum test with Benjamini Hochberg adjustment was used to determine significance. (C, D) Supervised analysis with sparse PLS-DA in ADHD (C) or subgroup (D) patients at the gene level. PERMANOVA calculation based on the Bray Curtis distance at the gene level.


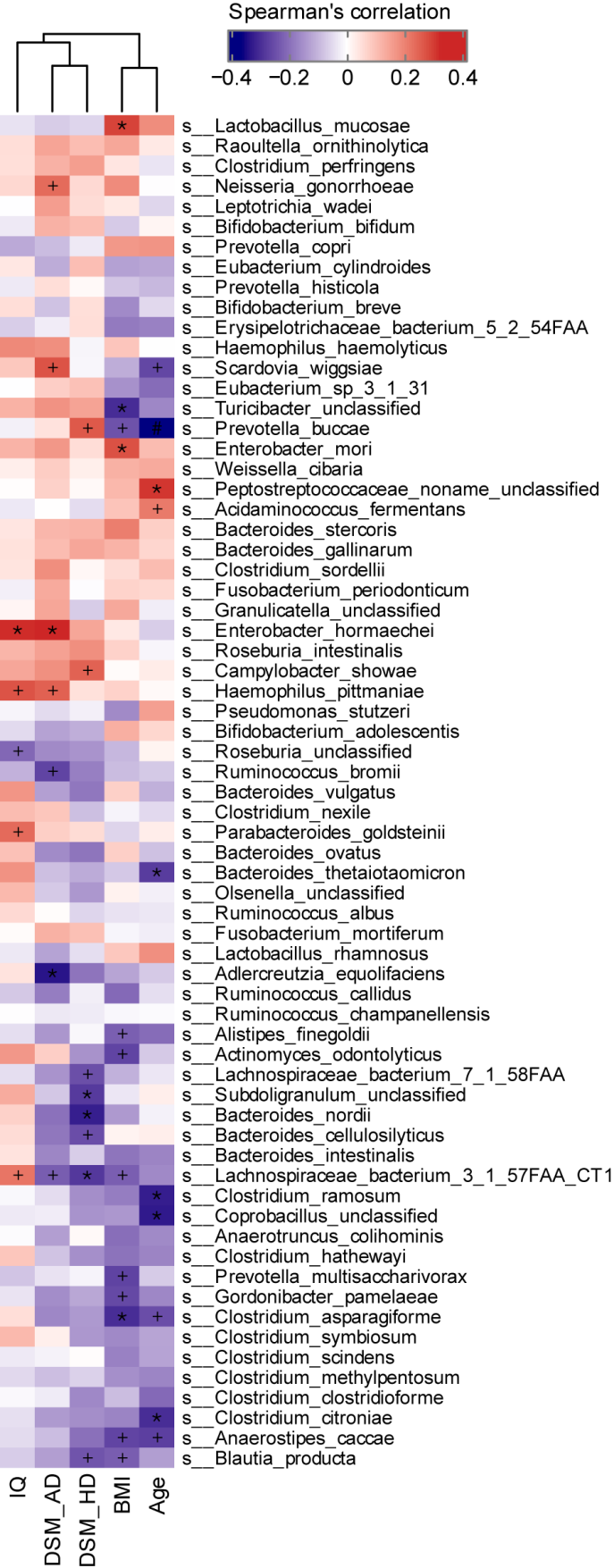


**Fig. S2 Correlation between gut microbiota species and clinical characteristics of ADHD patients (n = 91).** Species names were ordered according to Figure 4. Red and purple indicate positive and negative correlations, respectively. ‘+’ denotes *p* < 0.05; ‘*’ denotes *p* < 0.01.

**
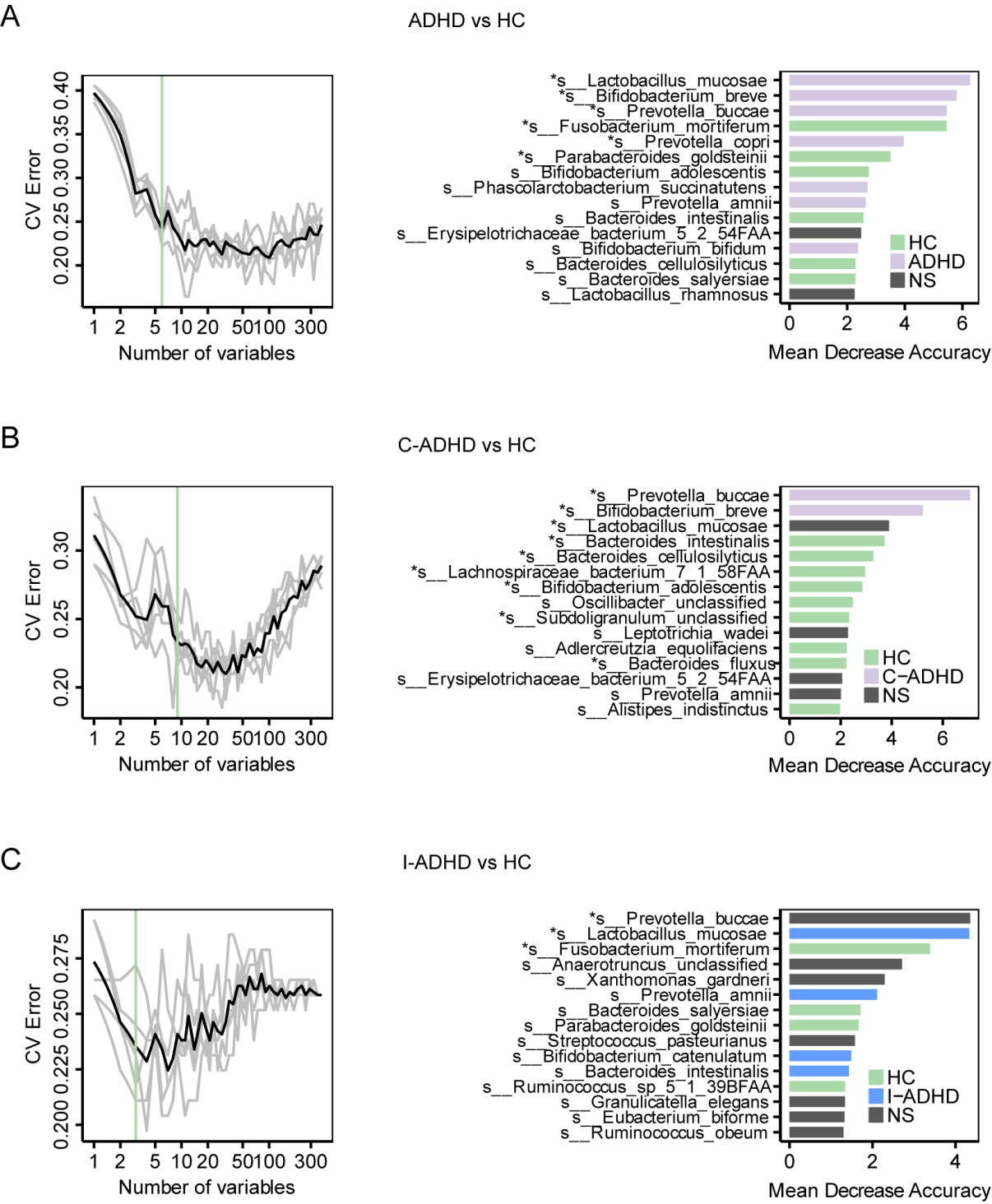
**

**Fig. S3 Selection of microbial markers to discriminate the total ADHD patient cohort and HCs (A), C-ADHD and HCs (B), I-ADHD and HCs (C) based on a random forest model.** Left panel: Distribution of 5 trials of 10-fold cross-validation error in random forest classification. The gray and black curves indicate the 5 trials and the average cross-validation error, respectively. The light green line represents the number of bacterial species in the classifier selected by the random forest model. Right panel: The top 15 most important species used to discriminate samples. The importance of each species was determined by using the mean decrease in accuracy. Bars were colored according to the enrichment, and gray indicates no significant difference. Species with asterisks indicate the species selected as the classifier by the random forest model.


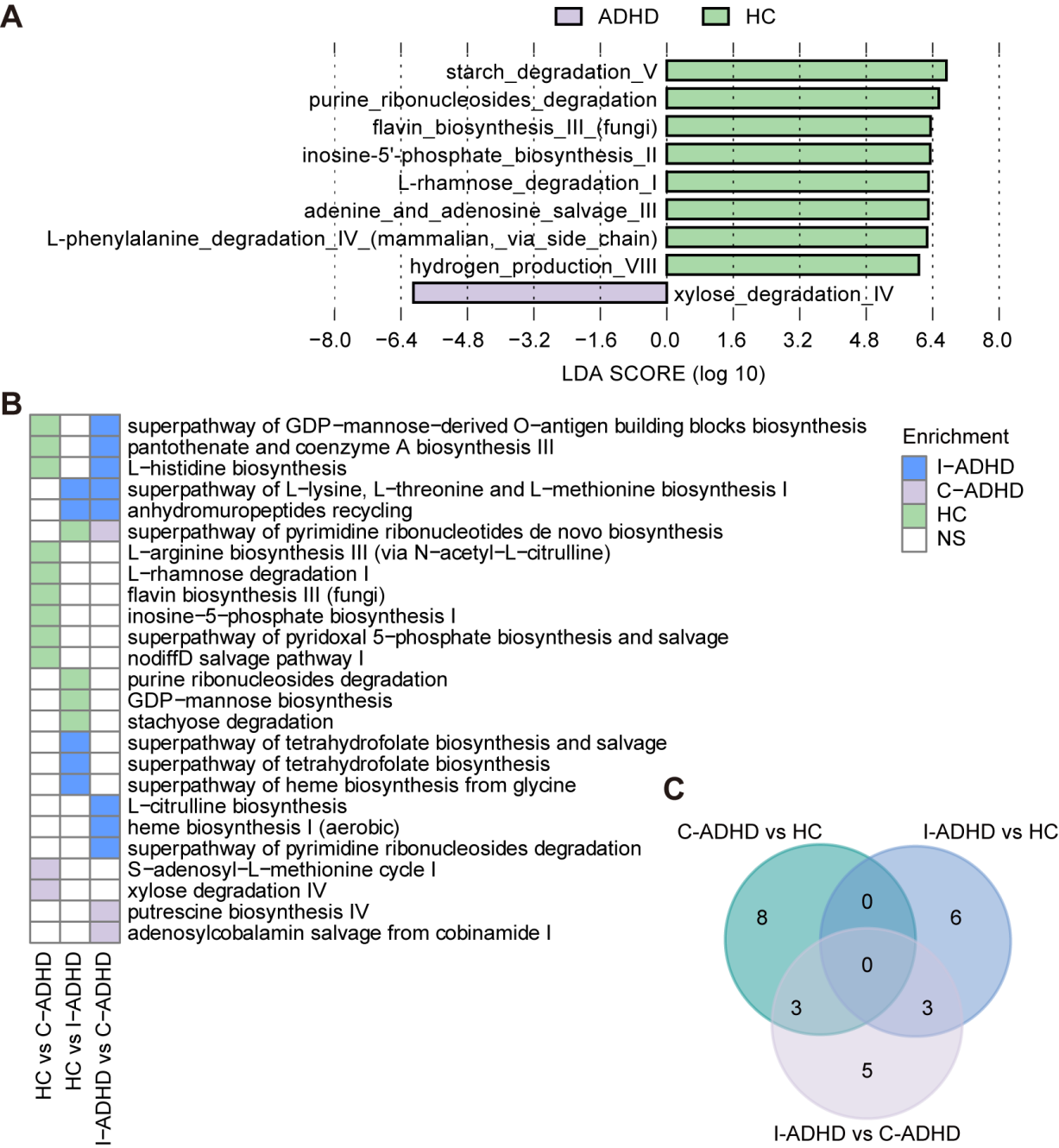


**Fig. S4 Gut microbial functions of C-ADHD patients are different from those of I-ADHD patients and HCs.** (A) Significantly different pathways between ADHD subgroup patients and HCs. The LDA scores (log 10) > 2 and *p* < 0.05 are shown. Bar length indicates the effect size of each species. (B) Heatmap of sharing significantly different pathways among different comparisons by LEfSe analysis. (C) Venn diagram of significantly different pathways among different comparisons.


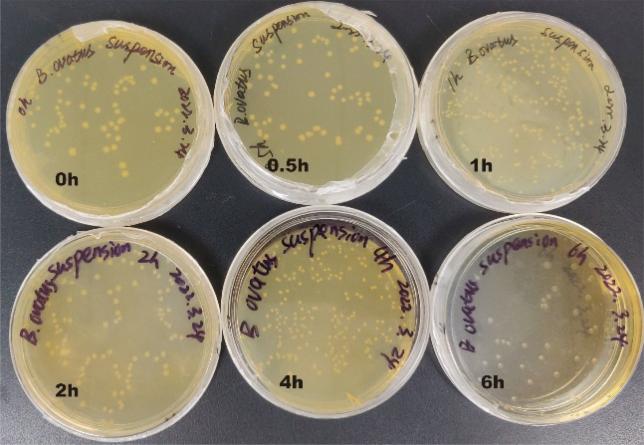


**Fig. S5 Verification of *Bacteroides ovatus* viability in drinking water.** The same amount of drinking water with *B. ovatus* was diluted 10^-6^ times at intervals of 0 h, 0.5 h, 1 h, 2 h, 4 h, and 6 h, and 100 µL diluent was carefully plated on solid thioglycolate medium and cultured under anaerobic conditions at 37°C. After 24 h, the growth of single colonies on the solid culture plates was checked.

**
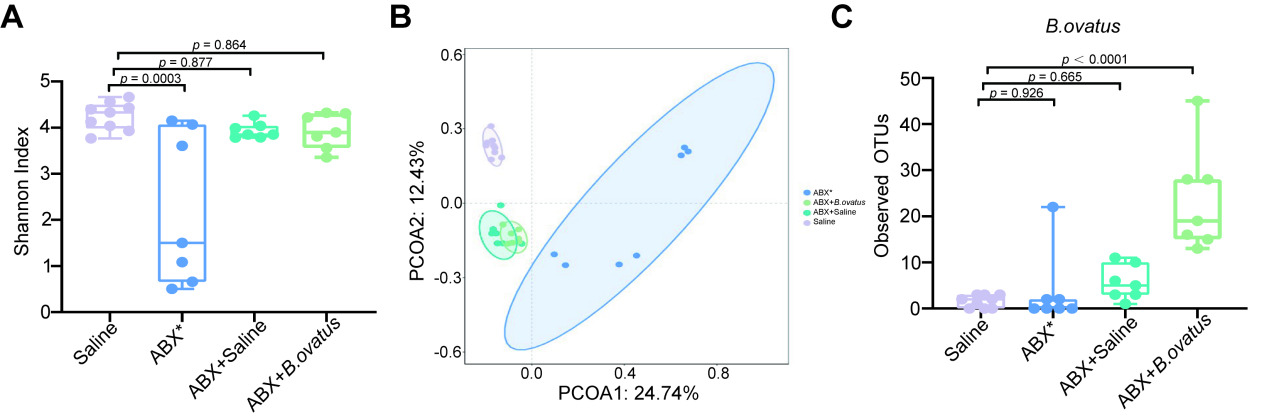
**

**Fig. S6 The gut microbiota changes in SHRs 14 days after bacterial transplantation.** (A) Shannon analysis of α diversity in gut microbiota. (B) Principal coordinates analysis (PCoA) plot of β diversity based on Bray–Curtis in different groups. (C) Observed operational taxonomic units (OTUs) of *Bacteroides ovatus* in different groups. Mean ± SEM are plotted; one-way ANOVA followed by the Tukey–Kramer post hoc test. Saline: treated with sterile saline in the whole process as a control, ABX*: after 10 days of antibiotic treatment in the ABX + saline group, ABX + saline: after 10 days of antibiotic and 14 days of saline treatment, ABX + *B. ovatus*: after 10 days of antibiotic and 14 days of *Bacteroides ovatus* ATCC 8483 gavage.

**
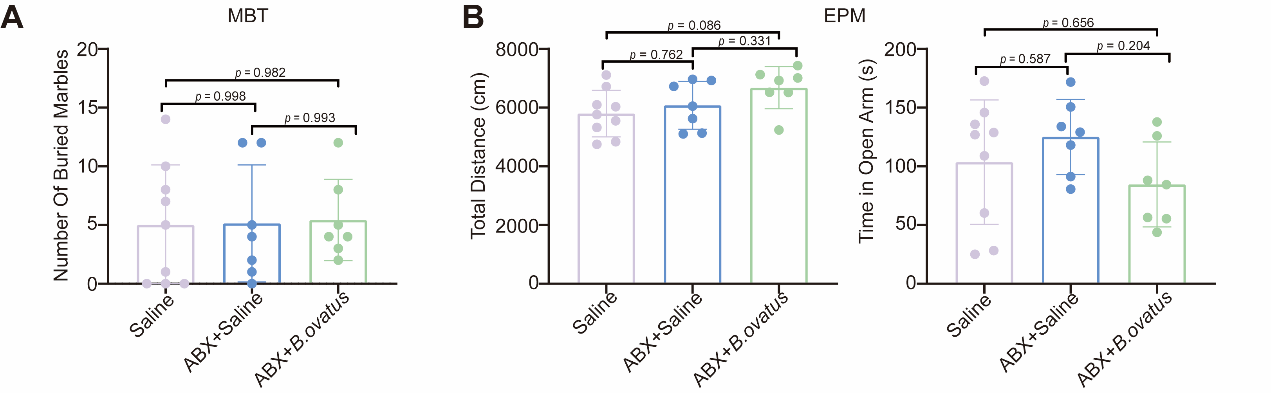
**

**Fig. S7 Anxiety behaviors were not altered in SHRs after *Bacteroides ovatus* ATCC 8483 gavage**. (A) The number of buried marbles in each group was recorded. (B) The total distance (left panel) and time spent on the open arm (right panel) of each group were compared in the EPM test. Mean ± SEM are plotted; one-way ANOVA followed by the Tukey–Kramer post hoc test. ABX: antibiotic cocktail, MBT: marble burying test, EPM: elevated plus maze.


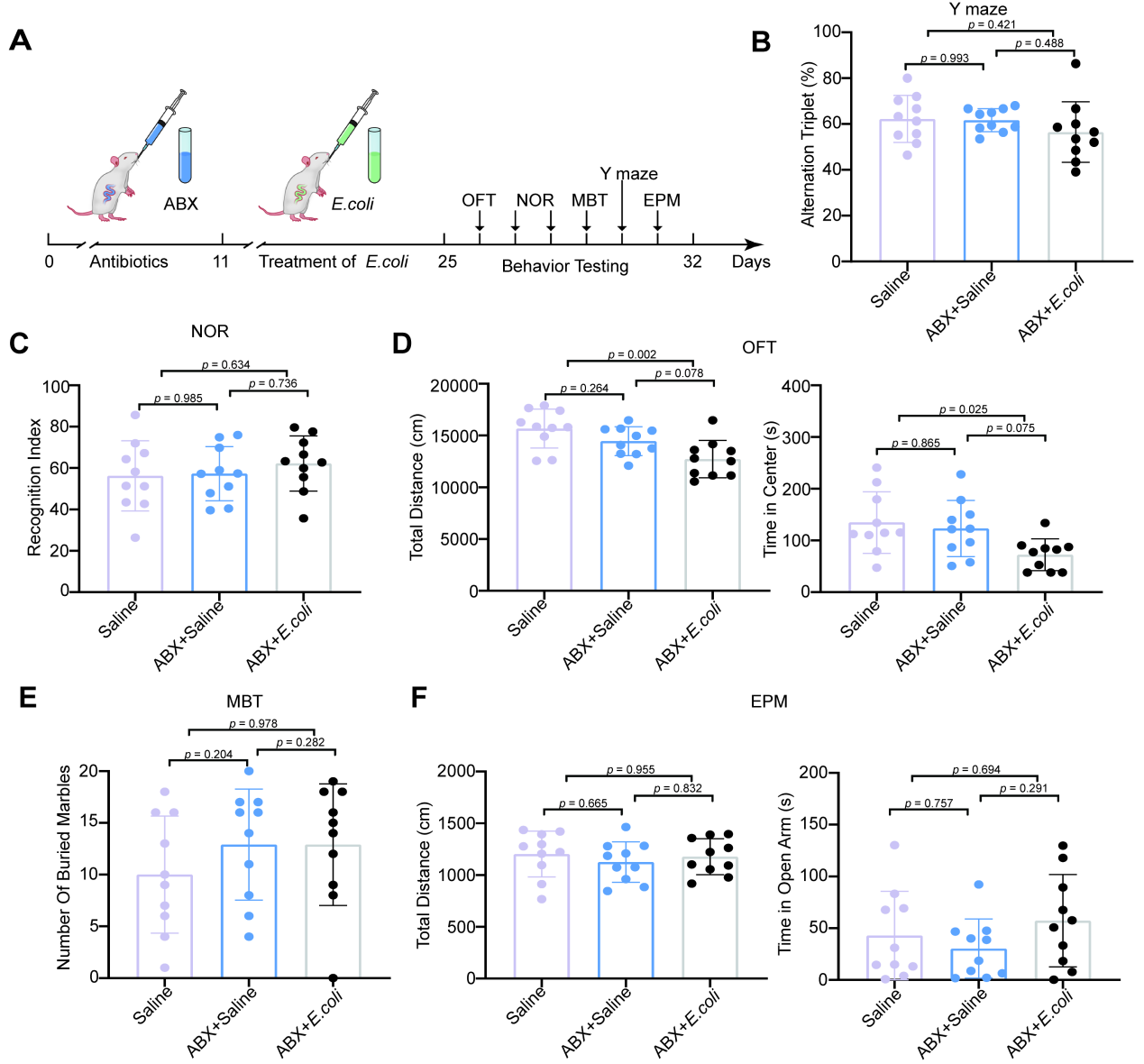


**Fig. S8 ADHD-like behaviors were not altered in SHRs after *Escherichia coli* AM12-30 gavage**. (A) Schematic diagram of ***Escherichia coli*** transplantation and behavioral tests. There were three groups (per group = 10 rats): Saline (gavage with saline during the first 24 days as a control), ABX+Saline (treated with an antibiotic cocktail within the first 10 days and saline for the next 14 days via oral gavage and drinking water), and ABX+*E. coli* (treated with an antibiotic cocktail within the first 10 days and *Escherichia coli* for the next 14 days via oral gavage and drinking water). (B) Spontaneous alternations of each group were recorded in the Y maze. (C) Comparison of the recognition index among different rat groups. (D) For the open field test, the time spent in the center and the total distance of locomotion of each group were analyzed. (E) The number of buried marbles in each group was recorded. (F) The total distance (left panel) and time spent on the open arm (right panel) of each group were compared in the EMP. Mean ± SEM are plotted; one-way ANOVA followed by the Tukey–Kramer post hoc test. ABX: antibiotic cocktail, OFT: open field test, NOR: novel object recognition, MBT: marble burying test, EPM: elevated plus maze.


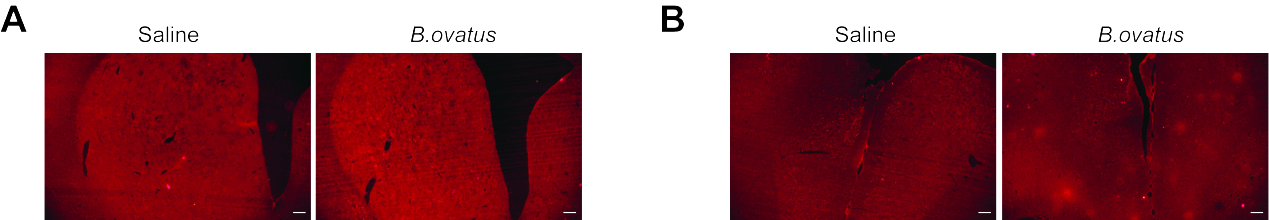


**Fig. S9 Administration of *Bacteroides ovatus* ATCC 8483 did not activate the neurons in the CPU or mPFC of SHRs.** Representative images of cFos staining in the (A) caudate-putamen (CPU) and (B) medial prefrontal cortex (mPFC) regions of SHRs 90 min after the Y maze test. Scale bar: 200 μm.

**Supplementary Tables**

**Table S1. Influence of host clinical characteristics on gut microbiota.**

|  | **PERMANOVA** | | | | **PERMDISP** | |
| --- | --- | --- | --- | --- | --- | --- |
| Phenotype | **Degree of freedom** | **R^2^** | p **value^a^** | Adjusted p value | p **value^a^** | Adjusted p value |
| Age | 1 | 0.00648 | 0.120 | 0.408 | 0.329 | 0.524 |
| Sex | 1 | 0.00883 | 0.021^*^ | 0.119 | 0.194 | 0.471 |
| BMI | 1 | 0.00545 | 0.266 | 0.678 | 0.019 | 0.136 |
| IQ | 1 | 0.01265 | 0.001^*^ | 0.017^*^ | 0.0001 | 0.002^*^ |
| Only child | 1 | 0.00456 | 0.495 | 0.765 | 0.262 | 0.512 |
| Cesarean section | 1 | 0.00498 | 0.379 | 0.742 | 0.169 | 0.471 |
| Premature birth | 1 | 0.00348 | 0.870 | 0.908 | 0.858 | 0.858 |
| Low birth weight | 1 | 0.00462 | 0.479 | 0.765 | 0.079 | 0.269 |
| Maternal pregnancy with metabolic disease | 1 | 0.00522 | 0.279 | 0.678 | 0.594 | 0.759 |
| Antibiotic application during pregnancy | 1 | 0.00397 | 0.710 | 0.862 | 0.053 | 0.225 |
| Antibiotic application in infant | 1 | 0.00336 | 0.882 | 0.908 | 0.789 | 0.853 |
| Infant feeding | 2 | 0.00875 | 0.632 | 0.826 | 0.339 | 0.524 |
| Side dishes preference | 3 | 0.01153 | 0.908 | 0.908 | 0.625 | 0.759 |
| Staple food preference | 3 | 0.02260 | 0.013^*^ | 0.111 | 0.803 | 0.853 |
| Yogurt preference | 1 | 0.00491 | 0.393 | 0.742 | 0.271 | 0.512 |
| Other fermented food preference | 1 | 0.00430 | 0.588 | 0.826 | 0.425 | 0.602 |

^a^The *p* values based on 9,999 permutations. **p* < 0.05

**Table** **S2 Dietary habits of the study cohort.**

| Characteristics | I-ADHD  (n = 38) | C-ADHD (n = 53) | HC  (n = 109) | **p** value^a^ |
| --- | --- | --- | --- | --- |
| Infant feeding, No. (%)  breast milk  formula milk  mixed feeding | 16 (42.1)  11 (28.9)  11 (28.9) | 21 (39.6)  16 (30.2)  16 (30.2) | 43 (39.4)  34 (31.2)  32 (29.4) | 0.999 |
| Side dishes preference, No. (%)  preference for meat  preference for vegetables  mixed meat and vegetables  vegetarian | 5 (13.2)  7 (18.4)  25 (65.8)  1 (2.6) | 11 (20.7)  9 (17.0)  32 (60.4)  1 (1.9) | 15(13.8)  14 (12.8)  79 (72.5)  1 (0.9) | 0.617 |
| Staple food preference, No. (%)  only refined grains  preference for refined grains  preference for whole grains  mixed refined and whole grains | 9 (23.7)  23 (60.5)  1 (2.6)  5 (13.2) | 14 (26.4)  32 (60.4)  1 (1.9)  6 (11.3) | 13 (11.9)  73 (67.0)  0 (0)  23 (21.1) | 0.064 |
| Yogurt preference, No. (%)  preference  little or never | 3 (7.9)  35 (92.1) | 9 (17.0)  44 (83.0) | 13 (11.9)  96 (88.1) | 0.461 |
| Other fermented food preference, No. (%)  preference  little or never | 3 (7.9)  35 (92.1) | 9 (17.0)  44 (83.0) | 7 (6.4)  102 (93.6) | 0.109 |

^a^*p* value based on Fisher’s exact test.

**Table S3 Defecation characteristics of the study cohort.**

| Characteristics | I-ADHD  (n = 38) | C-ADHD (n = 53) | HC  (n = 109) | **p** value^a^ |
| --- | --- | --- | --- | --- |
| Frequency, No. (%)  once more than two days  once or twice a day  more than three times a day | 6 (15.8)  29 (76.3)  3 (7.9) | 8 (15.1)  42 (79.2)  3 (5.7) | 11 (10.1)  91 (83.5)  7 (6.4) | 0.7724 |
| Smooth, No. (%)  basically smooth  habitual unsmooth defecate | 35 (92.1)  3 (7.9) | 44 (83.0)  9 (17.0) | 100 (91.7)  9 (8.3) | 0.241 |
| Shape, No. (%)  basically shaped  habitual shapeless | 38 (92.1)  0 (7.9) | 49 (92.5)  4 (7.5) | 107 (91.7)  2 (8.3) | 0.074 |

^a^*p* value based on Fisher’s exact test.

**Table S4 Relative abundances of different bacterial genera in the total ADHD patient cohort and HCs.**

| Genus | ADHD (mean ± S.D.) | HC (mean ± S.D.) | mean rank in ADHD | mean rank in HC | **p** value^a^ | Enrichment |
| --- | --- | --- | --- | --- | --- | --- |
| g__Prevotella | 10.8422 ± 23.6450 | 5.7004 ± 16.6239 | 121 | 89 | 0.0001* | ADHD |
| g__Subdoligranulum | 1.0550 ± 1.4859 | 1.5567 ± 2.0861 | 93 | 114 | 0.0158* | HC |
| g__Phascolarctobacterium | 0.0515 ± 0.3612 | 0.0478 ± 0.2903 | 94 | 113 | 0.0173* | HC |
| g__Adlercreutzia | 0.0392 ± 0.0812 | 0.0554 ± 0.0862 | 94 | 113 | 0.0177* | HC |
| g__Fusobacterium | 0.0257 ± 0.0937 | 0.2223 ± 1.1925 | 92 | 115 | 0.0065* | HC |
| g__Gemella | 0.0028 ± 0.0062 | 0.0031 ± 0.0045 | 93 | 114 | 0.0114* | HC |
| g__Scardovia | 0.0011 ± 0.0025 | 0.0007 ± 0.0018 | 113 | 96 | 0.0423* | ADHD |
| g__Methyloversatilis | 0.0006 ± 0.0013 | 0.0008 ± 0.0014 | 95 | 112 | 0.0216* | HC |
| g__Brevundimonas | 0.0001 ± 0.0005 | 0.0003 ± 0.0007 | 99 | 109 | 0.0432* | HC |

^a^*p* value based on the Wilcoxon rank-sum test. **p* < 0.05

**Table S5 Relative abundances of eukaryota and viruses between ADHD and HCs.**

| Taxa | ADHD (mean ± S.D.) | HC (mean ± S.D.) | mean rank in ADHD | mean rank in HC | **p** value^a^ |
| --- | --- | --- | --- | --- | --- |
| k__Eukaryota\|p__Ascomycota\|c__Saccharomycetes\|o__Saccharomycetales\|f__Saccharomycetaceae\|g__Saccharomyces\|s__Saccharomyces_cerevisiae | 0.0033 ± 0.0165 | 0.0013 ± 0.0079 | 105 | 104 | 0.8803 |
| k__Eukaryota\|p__Microsporidia\|c__Microsporidia_noname\|o__Microsporidia_noname\|f__Enterocytozoonidae\|g__Enterocytozoon\|s__Enterocytozoon_bieneusi | 0.0001 ± 0.0002 | 0.0001 ± 0.0004 | 100 | 108 | 0.1139 |
| k__Viruses\|p__Viruses_noname\|c__Viruses_noname\|o__Caudovirales | 0.0563 ± 0.3211 | 0.0219 ± 0.1323 | 107 | 101 | 0.2527 |

^a^*p* value based on Wilcoxon rank-sum test.

**Table S6 Relative abundances of different bacterial genera among I-ADHD, C-ADHD and HCs.**

| Genus | I-ADHD (mean ± S.D.) | C-ADHD (mean ± S.D.) | HC (mean ± S.D.) | mean rank in I-ADHD | mean rank in C-ADHD | mean rank in HC | **p** value^a^ | Enrichment |
| --- | --- | --- | --- | --- | --- | --- | --- | --- |
| g__Prevotella | 7.7403 ± 21.4784 | 14.3709 ± 26.1057 | 5.7004 ± 16.6239 | 107 | 124 | 87 | 0.0004* | C-ADHD |
| g__Roseburia | 1.9777 ± 2.6309 | 3.8243 ± 5.2360 | 2.9289 ± 3.2291 | 79 | 104 | 106 | 0.0447* | HC |
| g__Bifidobacterium | 3.5763 ± 3.3021 | 2.3468 ± 2.9024 | 2.6185 ± 2.5194 | 119 | 87 | 100 | 0.0357* | I-ADHD |
| g__Subdoligranulum | 1.4957 ± 1.7370 | 0.8076 ± 1.3133 | 1.5567 ± 2.0861 | 111 | 76 | 109 | 0.0014* | I-ADHD |
| g__Bilophila | 0.4680 ± 0.5646 | 0.2805 ± 0.4379 | 0.3801 ± 0.3711 | 109 | 83 | 106 | 0.0350* | I-ADHD |
| g__Oscillibacter | 0.6648 ± 0.9545 | 0.2475 ± 0.3046 | 0.4252 ± 0.4613 | 120 | 76 | 106 | 0.0007* | I-ADHD |
| g__Fusobacterium | 0.0179 ± 0.0628 | 0.0328 ± 0.1157 | 0.2223 ± 1.1925 | 87 | 88 | 111 | 0.0206* | HC |
| g__Adlercreutzia | 0.0612 ± 0.1194 | 0.0235 ± 0.0379 | 0.0554 ± 0.0862 | 100 | 81 | 110 | 0.0119* | HC |
| g__Acidaminococcus | 0.0384 ± 0.1357 | 0.0048 ± 0.0281 | 0.0071 ± 0.0276 | 124 | 91 | 97 | 0.0059* | I-ADHD |
| g__Listeria | 0.0000 ± 0.0001 | 0.0002 ± 0.0006 | 0.0000 ± 0.0001 | 93 | 113 | 97 | 0.0027* | C-ADHD |

^a^*p* value based on the Kruskal–Wallis test. **p* < 0.05
