# Supplementary material for: Gut metagenomic characteristics of ADHD reveal low Bacteroides ovatus-associated host cognitive impairment
Source: Gut Microbes. 2022 Sep 20;14(1):2125747. doi: 10.1080/19490976.2022.2125747 (PMC9519028; doi:10.1080/19490976.2022.2125747)

Gut metagenomic analysis for patients with ADHD aged 6-15 years

Group leader unit: Xijing Hospital

Project leader: Xiong Lize

Department: Anesthesiology

Contact number: 13720418610

Study period: From December 2017 to December 2022

Version: V2.0

Release date: December 18, 2017

**Research protocol**

**Name:** Gut metagenomic analysis for patients with ADHD aged 6-15 years.

**Background:** Attention-deficit/hyperactivity disorder (ADHD) is the most common mental disorder in child psychiatry. This study aims to provide new clues and directions for the diagnosis and treatment for ADHD by investigating gut microbiome and brain-gut axis.

**Research Period Planned time:** December 2017 to December 2022

**Contact:** liyanxjtu@xjtu.edu.cn;13720418610

**Sample source:** Fecal samples of ADHD patients in Xijing Hospital could be included without distinction of disease type and degree.

**Sample form:** The feces should be stored in the refrigerator at -80 °C and tested within one month.

**Expected number of samples:** 150 patients and 150 age-and sex-matched healthy controls, 300 cases in total.

**Inclusion criteria:** Patients aged 6-15 who meet the diagnostic criteria for ADHD in the Diagnostic and Statistical Manual of Mental Disorders IV (DSM-IV), and healthy children who matched the age of the children.

**Exclusion criteria:** Having other psychiatric disorders or neurological disorders; IQ below 70 on the Wechsler Intelligence Scale; history of treatment with any medication for ADHD; any antibiotic treatment for the least three months before sample collection.


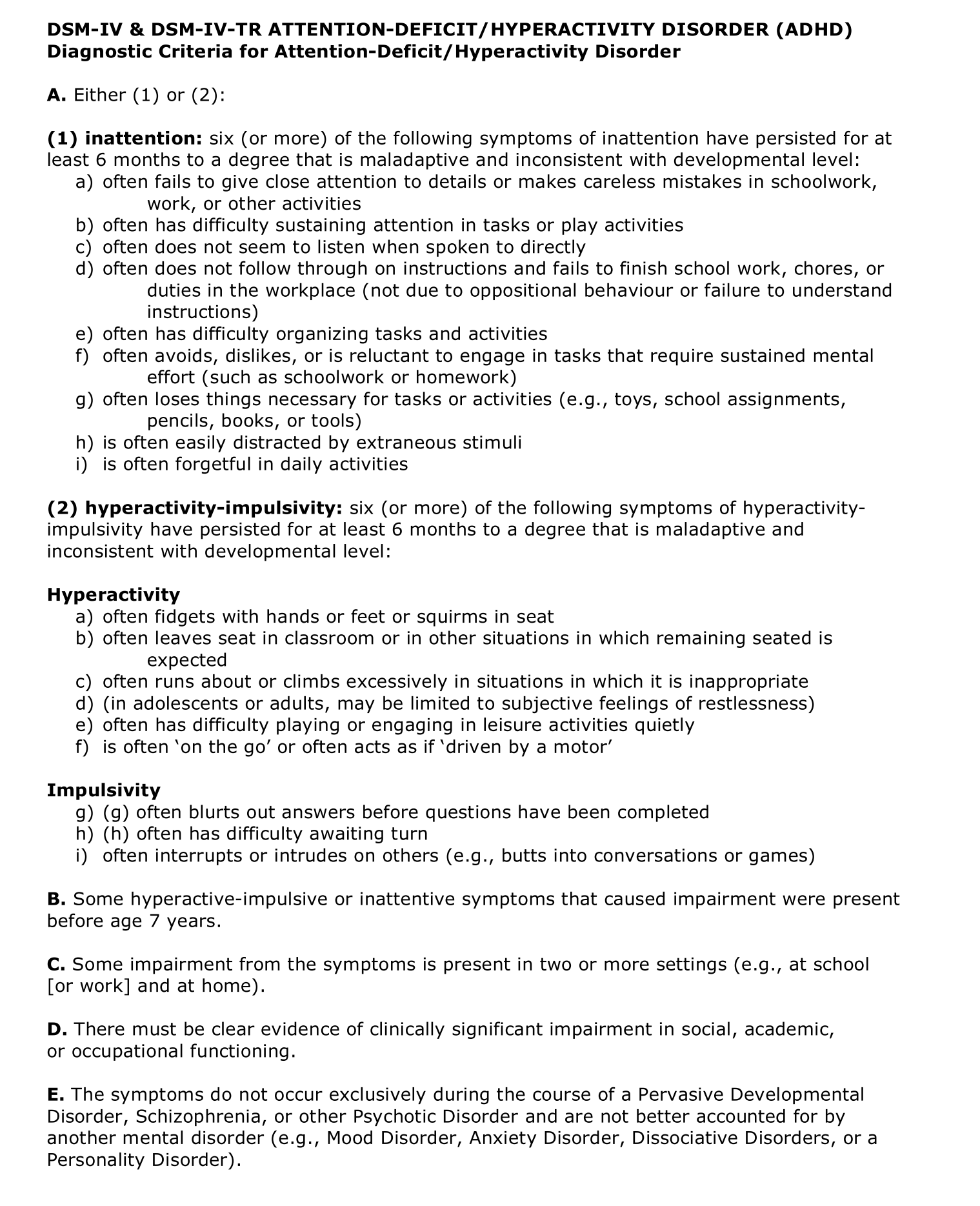


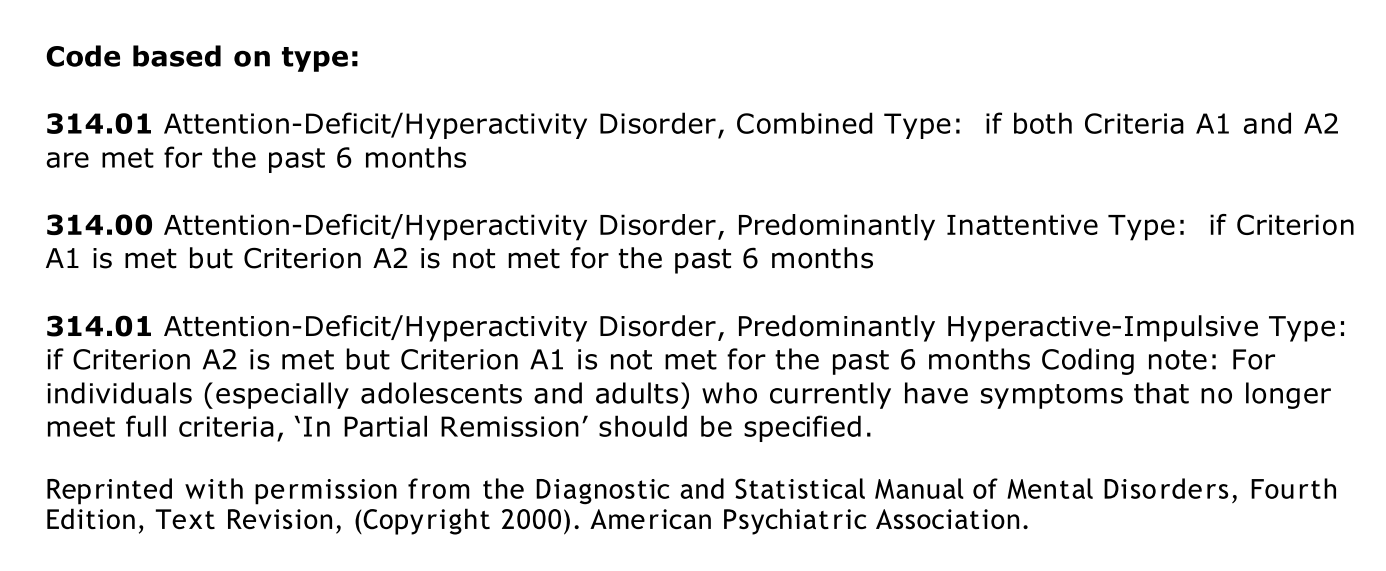


**Chinese Classification of Mental Disorders (CCMD-3)**

Attention-Deficit/Hyperactivity Disorder [F90.0]

ADHD occurs in childhood (mostly around age 3 years), and it is manifested as a group of syndromes with obvious difficulty in concentration, short duration of attention, and hyperactivity or impulsivity compared with individuals at a comparable level of development. Symptoms occurred in a variety of settings (e.g., at home, at school, or clinic) and were more common in boys than girls.

Diagnostic criteria for Attention-Deficit/Hyperactivity Disorder

**1. Inattention, at least have four of the following symptoms**

① easily distracted while studying and must to visit any extraneous stimulus

② very inattentive in class, often looking around or in a daze

③ often procrastinate and play while doing schoolwork, the schoolwork is dirty and messy, often do less or make mistakes

④ often fails to give close attention to details or makes careless mistakes in schoolwork, or other activities

⑤ often loses or particularly careless things (e.g., often makes a mess of clothes, books, etc.)

⑥ often does not follow through on instructions and fails to finish schoolwork, chores, etc.

⑦ often has difficulty sustaining attention in tasks and goes on to do other thing before finishing one thing

⑧ often does not seem to listen when spoken to directly

⑨ often loses things in daily activities

**2. Hyperactivity and impulsivity, at least have four of the following symptoms**

① often has difficulty sitting still or squirms in seat in situations in which remaining seated is expected

② often does the little trick in class, or plays with things or whispers with classmates

③ often talks a lot, interrupts, and blurts out answers before questions have been completed

④ is very noisy and often has difficulty playing quietly

⑤ often has difficulty observing order and discipline in collective activities (e.g., often rush to play in games and has difficulty awaiting turn)

⑥ often interferes with activities of others

⑦ often teases with children, easy to have disputes with classmates and not popular with peers

⑧ easily be excitable and impulsive with some excessive behavior

⑨ often runs about or climbs excessively in situations in which it is inappropriate, is adventurous and prone to accidents

[Severe Criteria] Adverse effects on social functioning (e.g., academic performance, interpersonal relationships, etc.).

[Criteria for disease course] Some symptoms that caused impairment were present before age 7 years (mostly around age 3 years), meeting symptom criteria and severity criteria for at least 6 months.

[Exclusion criteria] Mental retardation, pervasive developmental disorder, and mood disorder were excluded.

**Specific implementation:**

**1. Preparation**

Communicate with eligible subjects about the significance of the project and precautions for sample collection, and sign the informed consent after obtaining approval. At the early stage of collection, routine physical examination (height, weight) and questionnaire survey (health, dietary habits, history of other diseases, family history, medication history, etc.) can be conducted. Registration of the sample date, sample number, sample name, gender, age and other basic information. After registration, the following materials will be issued: One stool tray and 2 stool collectors with corresponding numbers will be issued according to the registration number.

**2.The collection of stool samples**

(1) Collection personnel: Subjects collect the data by themselves or assisted by their guardian.

(2) Material preparation: stool tray, stool collector, a reagent containing imidazolium-based ionic liquid, sterile cotton swab, sterile cryopreservation tube, sterile 30% glycerin solution, sterile water, portable refrigerator or ice box, label, pen, "Sample Registration Form" and liquid nitrogen tank.

(3) Collection quantity: 2 tubes, about 0.5-2 g for each tube.

(4) Collection steps:

A) The physician or nurse confirm that the patients and their guardian have signed the informed consent.

B) Check the sample number label of the subject, and explain to the patients and their guardian the purpose of retention and the method of cooperation, so as to obtain cooperation.

C) The patients and their guardian are instructed not to mix urine with feces during natural defecation. After obtaining stool samples, confirm that there is no other contamination (e.g., urine, etc.) in the stool tray.

D) With gloves on, the patients or their guardian shall immediately pick up the stool in the middle of the stool with the sampling spoon used for stool collection (as shown in the figure below), and avoid touching the part of the potty with the stool.


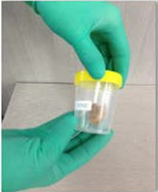


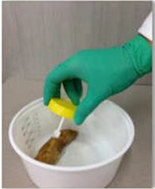


E) After the stool sample is placed in the collector, the cover shall be closed immediately and the study physician or nurse shall check the marked sample number.

F) A total of 2 sample collection tubes were collected, and each transfer was about 0.5-2 g (soybean-peanut size).

G) Temporarily store the fecal collector and fecal suspension in a portable refrigerator or ice box at 4°C, and freeze at -80°C as soon as possible

**3.Preservation of stool samples**

2 tubes of freshly collected stool samples were added to a reagent containing imidazolium-based ionic liquid, the samples were shaken and frozen in the -80 °C refrigerator within half an hour.

**4. Collect precautions**

(1) Remind subjects to collect enough fecal matter (at least one soybean size for each tube), and take the middle part of the fecal matter (puncture the middle part of the fecal matter with a fecal spoon, avoid taking the periphery of the fecal matter).

(2) The sample should be collected and put into the refrigerator within 30 minutes.

(3) Minimize the exposure time of fecal samples to the air.

(4) Normal and formed feces should be collected as far as possible.

**5. High-throughput fecal microbiome metagenomic sequencing**

Fresh fecal samples were collected, with 1 tube for microbiome high-throughput sequencing and 1 tube for backup storage.The fecal genome extraction kit was used to extract the total DNA from the samples. After purification and concentration determination, microbial metagenomic sequencing and diversity and community structure analysis were performed.

(1) Sequencing process:

**Flowchart of metagenomic research**

(2) Database construction sequencing process

A) Library construction strategy: 350 bp insert fragment library.

B) Sequencing strategy: The sequencing platform was X-TEN, PE101 or PE151 read length sequencing was used, and 5G clean data/ sample was recommended.


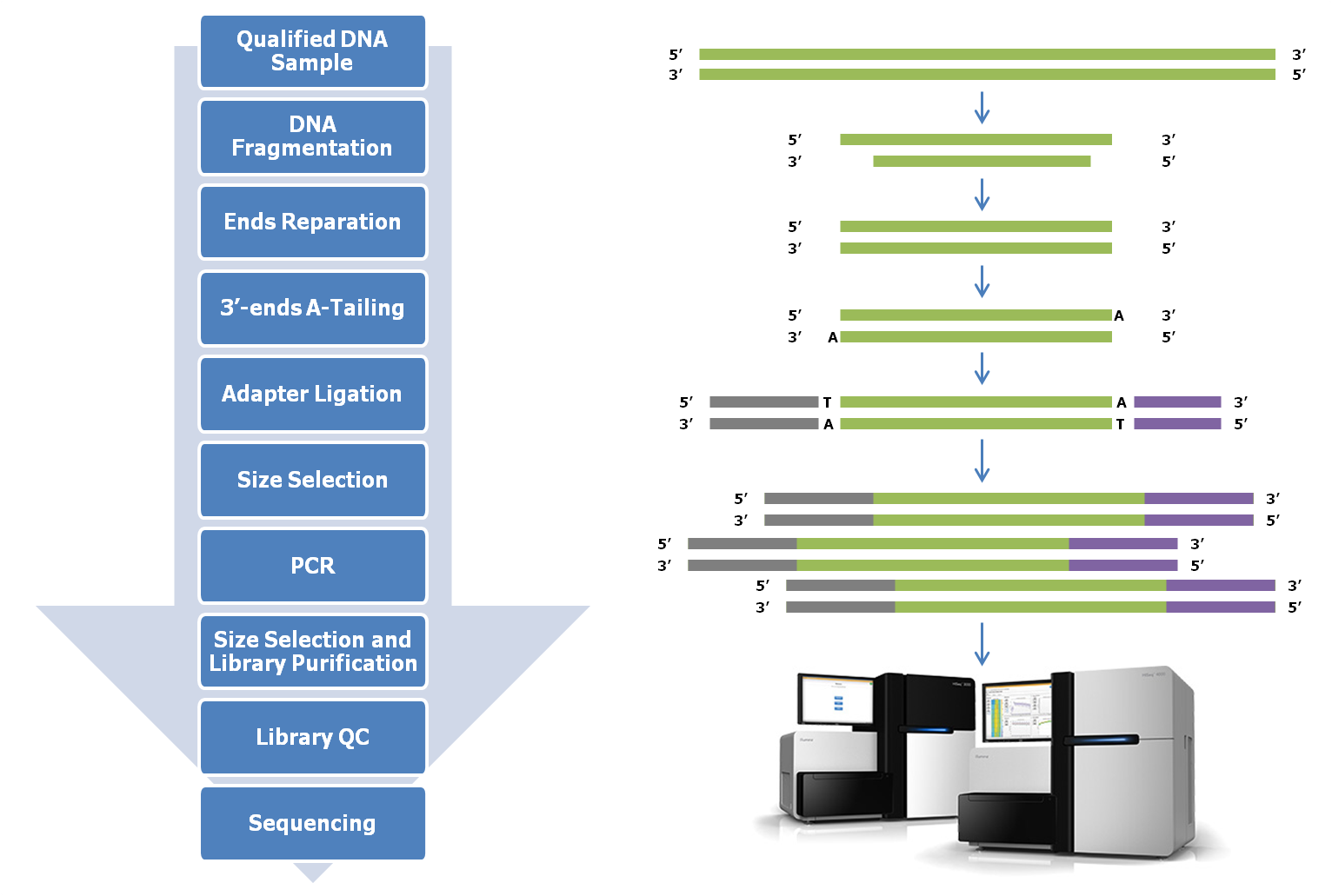


**Sequencing Flowchart of acer Genome Library (Hiseq)**

(3) Biological information analysis process

A) Sequencing and quality control: Clean data can be obtained from qualified offline data after quality control and filtration [1].

B) Assembly: If the human intestinal sample is directly compared with the human intestinal reference gene set ICG [2], otherwise, the non-human intestinal sample will be assembled using SOAPdenovo2[3] or IDBA[4] and other software for de Novo assembly to obtain the assembly results.

C) Gene and species annotation: Human intestinal samples will be directly compared with human intestinal reference gene set ICG to obtain gene set and its abundance, gene function and species annotation information; For non-human intestinal samples, the following analyses were performed to obtain relevant information: MetaGeneMark was used for genetic prediction of the assembly results to obtain the gene set [5]; The predicted genes were clustered using CD-HIT to get the non-redundant gene set [6]; The gene sets were annotated by comparing with public databases (including NR, Swiss-prot, COG, KEGG, GO, CAZy, eggNOG and ARDB) to obtain gene function and species annotation information.The Reads were compared with the back gene set, and the gene abundance and species abundance of each sample were calculated.Gene clustering: MLG, MGC or MGS and other methods are used to find gene sets of the same species or taxon [7].

D) Association analysis: unsupervised clustering: building the inherent pattern of data[8]; Multivariate statistics: Statistically significant correlation factors, such as disease status, healthy eating habits, blood sampling indicators, treatment differences and other factors, were analyzed by variable factor CCA/RDA or Spearman association analysis[9] - [13]; Difference test: Species diversity analysis and PCA analysis were conducted based on quantitative data of species abundance. Based on gene abundance, significant differences between groups were analyzed. Cluster heat map analysis was conducted based on significant difference genes. Obtain different species or genes between groups[9] - [13]; Functional analysis: Based on differential genes, GO and KEGG pathways were significantly enriched to identify differential metabolic pathways or modular units. Functional model: cross validation, select the best biomarkers related to sample classification, build a scalable classifier through cross validation, and predict new samples.

**reference**

[1] W. Li and A. Godzik, 'CP-Hit: A Fast Program for Clustering and Comparing Large Sets of Protein or Nucleotide sequences', Bioinformatics, Vol. 22, No. 13, pp. 1658 -- 1659, Jul. 2006.

[2] J. Li et al., 'An Integrated Catalog of Reference Genes in the Human Gut Microbiome', Nat. Biotechnol., Vol. 32, p. 834, 2014.

[3] R. Luo et al., SOAPdenovo2: An Empirically Improved MemoryEfficient Short-Read De Novo Assembler, Journal of Empirical Science, Vol. 1, No. 1, p. 18, Dec. 2012.

[4] Y. Peng, H. C. M. Leung, S. M. Yiu, and F. Y. L. Chin, 'Idba-ud:A de Novo Assembler for Single-cell and Metagenomic sequencing Data with Highly uneven Depth. ', Bioinformatics, Vol. 28, No. 11, pp. 1420 -- 1428, Jun. 2012.

[5] J. Qin et al., 'A Human gut microbial gene Catalogue Established by Metagenomic sequencing.' Nature, Vol. 464, No. 7285, pp. 59 -- 65, Mar. 2010.

[6] H. B. Nielsen et al., 'Identification and Assembly of Genetic Elements in Complex Metagenomic samples without Using Reference Genomes.', Nat Biotechnol, Vol. 32, No. 8, pp. 822 -- 828,Aug. 2014.

[7] J. Wang and H. Jia, 'Metagenome-wide Association Studies: Fine-mining the Microbiome.', Nat Rev Microbiol, Vol. 14, No. 8, pp. 508 -- 522, 2016.

[8] P. I. Costea et al., 'Landscape microbial community composition of Gut Microbial communities', Nat Microbiol, Vol. 3, No. 1, pp. 8-16, Jan. 2018.

[9] J. Qin et al., 'A Metagenome-wide Association Study of Gut Microbiota in Type 2 Diabetes.' Nature, Vol. 490, No. 7418, pp. 55-60, Oct. 2012.

[10] M. Whiteley, S. P. Diggle, and E. P. Greenberg, 'Progress in and Promise of Bacterial Quorum Sensing Research', Nature, Vol. 551, P. 313, Nov. 2017.

[11] D. Zeevi et al., 'Personalized Nutrition by Prediction of Glycemic Responses.', Cell, Vol. 163, No. 5, pp. 1079-1094, Nov. 2015.

[12] X. Zhang et al., 'The Oral and Gut Microbiomes are Perturbed in Rheumoid Arthritis and Partly Normalized After Treatment.', Nat Med, Vol. 21, No. 8, pp. 895 -- 905, Aug. 2015.

[13] Z. Jie et al., "The Gut Microbiome in Atherosclerotic cardiovascular Disease", Nat. Commun., Vol. 8, p. 845, 2017.

Signature of Principal Investigator:


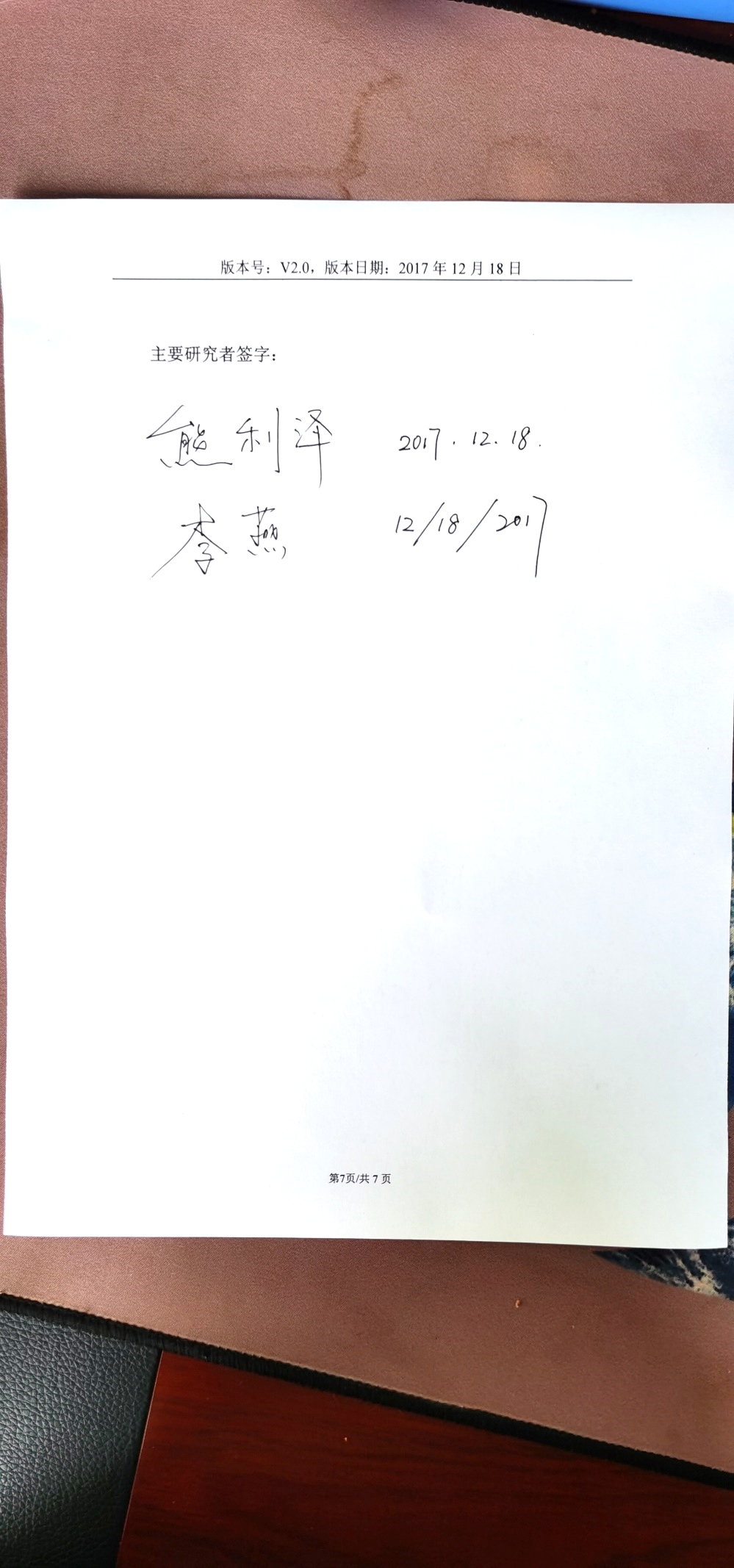

Supplement: Supplemental Material [file KGMI_A_2125747_SM1185.zip › 4 Supplemental material 2 Clinical Trial Protocol 20220519.docx]
