# Supplementary material for: Gut metagenomic characteristics of ADHD reveal low Bacteroides ovatus-associated host cognitive impairment
Source: Gut Microbes. 2022 Sep 20;14(1):2125747. doi: 10.1080/19490976.2022.2125747 (PMC9519028; doi:10.1080/19490976.2022.2125747)
Supplement: Supplemental Material [file KGMI_A_2125747_SM1185.zip › 5 Supplemental material 3 Patient informed consent 20220519.docx]

**PATIENT INFORMED CONSENT**

I agree to participate in this clinical trial, and declare and promise the following:

1. The procedure has been fully explained to me and I fully understand the nature of the procedure and technical requirements of this test in detail, including:
2. The aim of the present clinical trial is to differentiate the gut microbiota between patients with ADHD and healthy controls;
3. The test is free of charge from the sampling to the analysis of gut microbiota after the collection of fecal samples;
4. Precautions before and after collecting fecal samples;
5. Risk description: This test does not provide any additional diagnostic and therapeutic treatment for patients, and patients will not be at additional risk because of this test.
6. I make the following commitments:
7. Fill out a ***health and dietary habits questionnaire***;

(2) Cooperate with doctors to obtain qualified stool samples;

(3) Follow up regularly as required.

1. Voluntary participation and withdrawal

The hospital and the doctor are obliged to provide me with information related to this test and explain my concerns to me. Then I will voluntarily decide whether to participate or not. I have the right to withdraw at any stage and my withdrawal will not affect my routine treatment.

1. Confidentiality

The results and data obtained by this test belong to the project implementer and medical institutions and are free to use. My personal data will be kept confidential by the hospital. The ethics Committee of Xijing Hospital and the implementer can access the personal data. My identity will not be disclosed unless required by law. The results will be published for scientific purposes without revealing the identity of the individual.

1. Benefit

I will be examined and treated by experienced doctors. In order to fully protect my rights and interests, the hospital formulated a detailed test plan, which has been approved by the hospital ethics Committee.

1. Contact

The ethics committee of Xijing Hospital: 029-84771794; Department of Pediatrics, Xijing Hospital: 15398084750.

Signature-Patient:

Signature-Guardian:

Signature-Doctor:

Date:
