# Supplementary material for: Gut metagenomic characteristics of ADHD reveal low Bacteroides ovatus-associated host cognitive impairment
Source: Gut Microbes. 2022 Sep 20;14(1):2125747. doi: 10.1080/19490976.2022.2125747 (PMC9519028; doi:10.1080/19490976.2022.2125747)
Supplement: Supplemental Material [file KGMI_A_2125747_SM1185.zip › 6 Supplemental material 4 Health and dietary habits questionnaire 20220519.docx]

**Health and dietary habits survey**

Now we make a simple questionnaire survey on your basic information, including: height, weight, defecation, eating habits, and antibiotic use. (Please fill in the box with the capital letters of the option number according to your own health status for the corresponding question.)

**Name: Gender:** □ Male;□ Female **Date of birth: Nationality:**

| Height: cm | | Weight: kg | |
| --- | --- | --- | --- |
|  | Pick | The options | |
| Gestational age at birth |  | A.≥22 weeks to < 28 weeks; B.≥28 weeks to < 37 weeks;  C.≥37 weeks to < 42 weeks;D. 42 weeks or more | |
| Birth weight |  | A. < 2 kg;B. ≥ 2kg to < 3kg;C. ≥ 3kg to < 5kg;  D. ≥ 5kg to < 8kg; E. ≥ 8 kg | |
| Brothers and sisters |  | A. 1; B. 2; C. 3; D. 4; E. > 4 (___) | |
| Mode of production |  | A. natural birth; B. cesarean section | |
| Infant diet |  | A. mainly breast milk; B. mainly formula milk; C. mixed feeding | |
| Maternal metabolic disease during pregnancy? |  | 1.yes (A. diabetes; B. Hypertension; C. others __________);  2. no | |
| Does the mother use antibiotics during pregnancy? |  | 1.yes (name _________ delivery methods ______) 2.no | **See the last two columns** |
| Are antibiotics used during the neonatal period? |  | 1.Yes (name _________ delivery methods ______) 2.no |  |
| Stool frequency |  | A. once every 2 days; B.1-2 times/day; C.3-5 times/day; D. > 5 times/day | |
| Stool smooth |  | A. basically smooth; B. habitual unsmooth defecate | |
| Stool shape |  | A. basically shaped; B. habitual shapeless | |
| Other medical history |  | A. yes. (name _________ ); B. No. | |
| Diet |  | A. balanced meat and vegetable; B. meat-based;  C. mainly vegetarian; D. completely vegetarian (vegetarian) | |
| Staple food structure |  | A. rice/noodles mainly, a small amount of whole-grain or potato;  B. rice/noodles, whole-grain, and potato are basically equal in quantity;  C. whole-grain/potato mainly, a small amount of rice or noodles;  D. only rice/ noodles, and basically do not eat whole-grain or potato | |
| Have you had any probiotic drinks (e.g.Yakult) or probiotic drugs within the last 3 months? |  | A. yes. (name _________ ); B. no | |
| The frequency of intaking probiotic beverages (e.g. Yakult) |  | A. drink every day; B. drink > 3 times a week;  C. drink less than 2 times a week; d. Hardly | |
| Do you often eat fermented bean curd? |  | A. eat regularly; B. 2-3 times per week; C. occasionally; D. hardly | |
| Do you often eat pickles/sauerkraut? |  | A. eat regularly; B. 2-3 times per week; C. occasionally; D. hardly | |
| Often eat bean paste kind of food? |  | A. eat regularly; B. 2-3 times per week; C. occasionally; D. hardly | |
| Have you been on antibiotics for the last three months? |  | A. yes; B. no | |
| How these antibiotics were administrated? |  | A. oral; B. intravenous injection; C. external ointment; D. facial applications (nasal drops, ear drops, eye drops) | |
| Have you had any of the following antibiotics in the last 3 months? If not found in the table, please fill in the "other" column directly. | □azithromycin □cefaclor □cefixime □amoxicillin □ceftriaxone  □cefoperazone □meropenem □cilastatin □ethylsuccinate  □amoxicillin/penicillin (penicillin) □vancomycin □ampicillin  □piperacillin □cephalosporin □cefazolin □cephalosporins □amikacin □gentamycin □neomycin □streptomycin □clarithromycin  □erythromycin □tetracycline □oxytetracycline □minocycline  □doxycycline □ciprofloxacin □ofloxacin □moxifloxacin □norfloxacin  □mafenide □sulfacetamide and □other _________________________  __________________________________________________ | | |
